# How Ti Doping Improves the Catalytic Methane Dry Reforming of Nanoporous Reduced LaNiO_3_ Perovskites

**DOI:** 10.1021/acsanm.5c04175

**Published:** 2025-11-06

**Authors:** Thomas F. Winterstein, Andreas Oss, Christoph Malleier, Asghar Mohammadi, Bernhard Klötzer, Stefan Stöber, Volker Kahlenberg, Simon Penner

**Affiliations:** † Institute of Physical Chemistry, 27255 University of Innsbruck, Innrain 52c, Innsbruck A-6020, Austria; ‡ Institute of Mineralogy and Petrography, 27255 University of Innsbruck, Innrain 52d, Innsbruck A-6020, Austria; § Institut für Geowissenschaften Und Geographie, Martin-Luther Universität Halle, Von-Seckendorff − Platz 3, Halle (Saale) D-06120, Germany

**Keywords:** single perovskite, Ti-doped LaNiO_3_, in situ, La_2_Ti_2_O_7_, thermogravimetry, X-ray diffraction

## Abstract

We studied the effect of Ti substitution on nickel B-sites in LaNiO_3_ to unravel the influence of Ti doping on structural stability, Ni exsolution, and methane dry reforming (DRM) properties. Ni can be substituted by Ti down to compositions of x_Ni_ = 0.25 without compromising both phase and structure purity. At even higher Ti doping levels, formation of the pyrochlore-type La_2_Ti_2_O_7_ phase occurs. Ti substitution has a significant influence on the stability under reducing conditions and the appearance of specific intermediate structures relevant for DRM operation. Full decomposition is only observed for LaNiO_3_ and the x_Ni_ = 0.75 sample, which yield the La_2_O_3_ phase relevant for DRM activity at low Ti doping levels. A common impurity phase between x_Ni_ = 0.75 and 0.25 is La_2_TiO_5_, which acts as a Ti and La sink and hinders the formation of La_2_O_3_. For higher Ti doping levels, hydrogen reduction increases the amount of La_2_Ti_2_O_7_. A common denominator of all samples after hydrogen reduction is the full leaching of all nominally available Ni from the perovskite. The self-activation properties during DRM operation strongly depend on the Ti substitution level. Self-activation with either full or partial decomposition is only possible for LaNiO_3_ and x_Ni_ = 0.75, where intermediate lanthanum oxycarbonate formation occurs. For x_Ni_ = 0.50, the remaining perovskite structure is stable, but Ni exsolution nevertheless occurs, triggering DRM activity. Successive Ti doping invokes a change in the DRM mechanism from oxycarbonate-based at low Ti amounts (LaNiO_3_ and x_Ni_ = 0.75) to a more reactive-oxygen—dominated one for samples x_Ni_ ≤ 0.50, as indicated by X-ray photoelectron spectra. Ti doping also allows to economize the amount of Ni for DRM applications— it can be lowered to a quarter of the initial amount referenced to pure LaNiO_3_ without compromising DRM activity.

## Graphical abstract

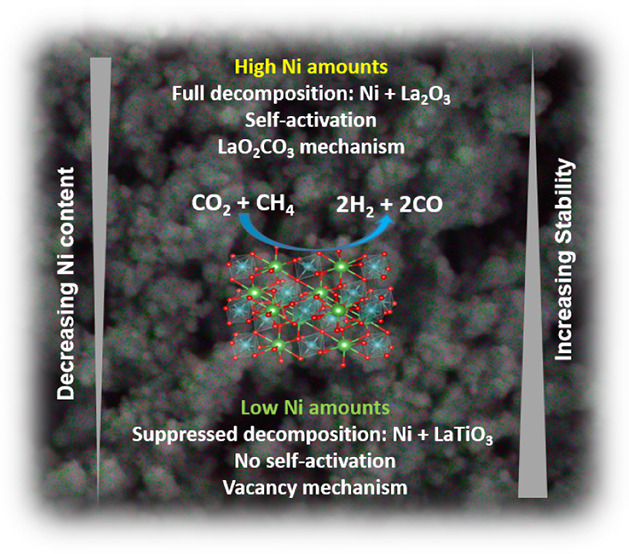

## 1 Introduction

Methane dry reforming (DRM) is a viable pathway to convert two harmful greenhouse gases into useful syngas (i.e., H_2_/CO) mixture, which can be subsequently used to tailor the synthesis of a variety of different synthetic chemicals. The DRM reaction (eq 1)­

1 $$CO_{2}+CH_{4}⇆2CO+2H_{2}$$

is highly endothermic, requiring high reaction temperatures between 600 and 800 °C to activate both carbon dioxide and methane. The main DRM reaction is connected to several accompanying side reactions in a complex reaction network relevant for both catalyst regeneration and selectivity spoiling reactions.^1–3^ Current catalyst development is directed toward lowering the reaction temperature and overcoming the limitations of severe coking and particle sintering at the high reaction temperatures of the commonly used catalyst material, metallic nickel.^4,5^ To overcome these restrictions, perovskite materials have evolved in the last two decades as promising materials to deal with the detrimental properties of the archetypical nickel-based materials.^2^ Perovskites are complex oxides of the general formulation ABO_3_, where A are typically 2- or 3-valent metal ions of the earth alkaline group of metals or lanthanum. B-site ions are usually transition metal ions with multiple oxidation states, whose selection is mainly adapted to the specific catalytic use.^2^ One particular advantage of such perovskites is that their structural stability not only allows to tune their physicochemical properties, but as a consequence also to use them as precursor materials for steered decomposition into a catalytically active metal-oxide system.^6–8^ This concept is strongly tied to the approach of metal exsolution from the perovskite host structure.^9–13^ With this approach, a stronger anchoring of the exsolved metal particles to the supporting oxide usually allows controlling the morphology and size of the exsolved metal particles and enhancing the sintering stability. However, despite this apparent advantage, it involves structurally transferring a much simpler material (i.e., the perovskite) into a much more complex system (i.e., a metal-oxide system). As a structural consequence, a metal-oxide phase boundary is created. Catalytically, a bifunctionally operating mechanism is usually prevalent. For DRM applications, LaNiO_3_ serves as a highly illustrative example. Exsolution, typically in hydrogen or directly in the DRM mixture, transfers LaNiO_3_ into a Ni–La_2_O_3_ composite – methane is activated on metallic Ni, carbon dioxide on La_2_O_3_ via reversible formation of lanthanum oxycarbonate.^6^ This example, however, is only a benchmark material, where full decomposition of the perovskite precursor is achieved. In most cases, especially upon direct activation during DRM, only partial decomposition creates an even more complex structural mix consisting of exsolved metal particles, remnants of the initial perovskites and oxide-related decomposition products. The vast majority of literature-reported cases belongs to the latter group.^14^ Inevitably, it is close to impossible to infer the inherent activity of such metal-perovskite/oxide interfaces, let alone derive mechanistic details. The need for pathways to structure- and phase-pure metal-perovskite or – oxide interfaces is therefore imperative.

We have recently reported that such a pathway is feasible in the lanthanum–nickel-titanate perovskite phase diagram.^15^ Starting from different single- and double perovskite structures we were able to create specfiic interfaces of Ni and La_2_NiTiO_6_, single Ti-doped LaNi_0.5_Ti_0.5_O_3_ and La_2_TiO_5_/LaTiO_3_ for fixed Ni/Ti composition and to reveal their inherent DRM activity alongside structural consequences of interface formation. Such nickel titanate perovskites were studied in hydrocarbon dry reforming,^3,16,17^ methane partial and steam reforming,^18,19^ carbon dioxide and bireforming of methane^18,19^ or ethanol steam reforming.^20^ For dry reforming applications, Ni doping into Ca_0.8_Sr_0.2_TiO_3_ caused in situ formation of a Ni-perovskite interface with high DRM activity and easy reverse oxygen spillover to Ni.^16^ Sr-doped La_0.6_Sr_0.2_Ti_0.85_Ni_0.15_O_3_ perovskites were revealed to benefit from introduction of Ti to increase the number of exsolved Ni particles, as well as improving the catalyst stability. Similarly, the addition of Ti to La_0.46_Sr_0.34_Ti_0.9_Ni_0.1_O_3_ caused suppressed coke deposition due to a smaller particle size of Ni^0^ produced by reduction of Ni within the perovskite phase.^17^

As recent studies for a single perovskite with LaNi_0.5_Ti_0.5_O_3_ stoichiometry revealed an outperformance of undoped LaNiO_3_ in DRM after a hydrogen activation–DRM cycle with improved long-term catalytic activity, the present contribution systematically threads in the La_2_O_3_–NiO-TiO_2_ phase diagram to reveal inherent structural differences in Ni-perovskite interface formation with the potential to further economize the amount of Ni by maintaining high DRM activity and stability. Such an approach has been recently also shown to be a feasible pathway to decrease the amount of noble metals in copper-based perovskites for deNO_*x*_ applications.^21^

## 2 Experimental Section

### 2.1 Synthesis of Materials

The LaNi_*x*_Ti_1–*x*_O_3_ perovskite materials were obtained by a modified Pechini method.^15^ Stoichiometric amounts of La­(NO_3_)_3_ · 6 H_2_O (Carl Roth, 99.99%) and Ni­(NO_3_)_2_ · 6 H_2_O (Puratronic, 99.9985%) were dissolved in a citric acid solution with an ionic ratio of La+Ni+Ti: citric acid = 1:2. The resulting mixture was heated to and subsequently continuously stirred at 60 °C until all starting materials dissolved. Stoichiometric amounts of titanium-isopropoxide (Sigma-Aldrich, 97%), as well as ethylene glycol with an ionic ratio of La+Ni+Ti: ethylene glycol = 1:4, were added to the solution. The combined mixture was heated to 140 °C at a rate of 10 °C min^–1^ and then stirred for 3 h until a dry resin formed, which was subsequently calcined in a muffle furnace in air at 450 °C for 4 h. The resulting ashes were ground by hand in an agate mortar to a powder and sintered in air at 800 °C for 17 h. After sintering, the sample was ground up again, resulting in a black to light green homogeneous powder. Three g each of nine products were synthesized with nominal compositions of LaNi_*x*_Ti_1–*x*_O_3_ [x_Ni_ = 1; 0.75; 0.5; 0.25; 0.2; 0.15; 0.1; 0.05; 0]. The amounts of the starting materials for each synthesis can be found in Table S1. The program VESTA^22^ was employed to visualize the respective crystal structures (Figure 1).

**Figure 1:**
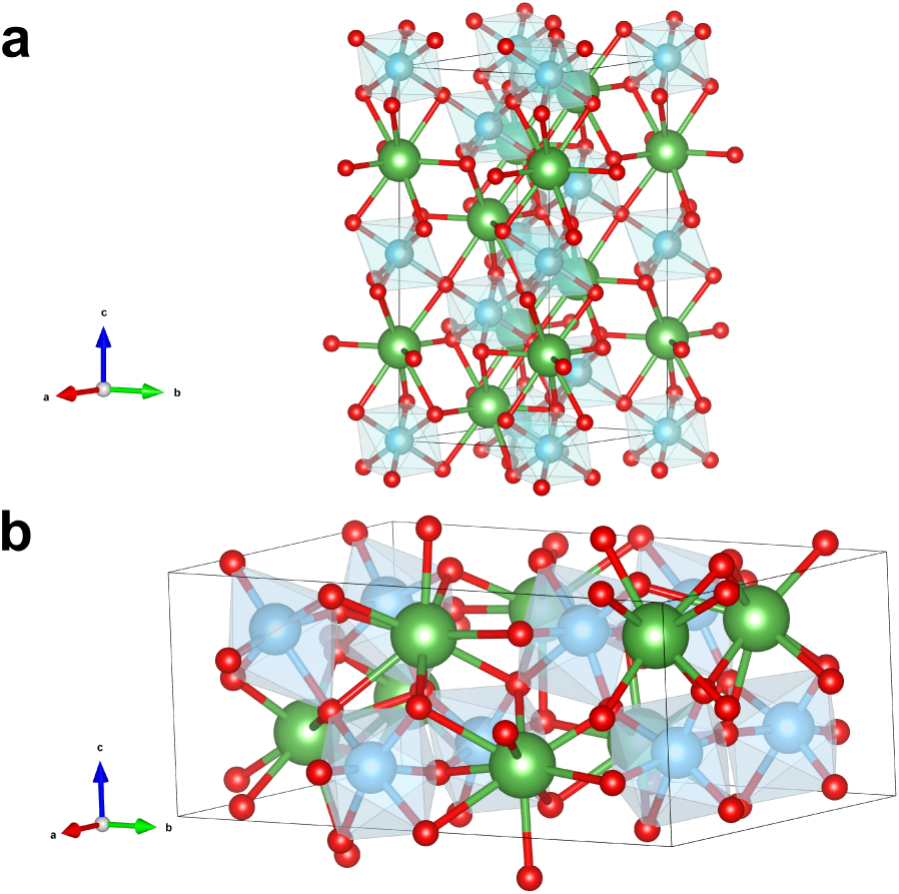
Unit cells of Panel **a** perovskite-type La­(Ni,Ti)­O_3_ ^24^ and Panel **b** pyrochlore-type La_2_Ti_2_O_7_ ^25^ using the VESTA program.^22^

### 2.2 X-ray Fluorescence Analysis (XRF)

A WDXRF S8 Tiger Series 2 (Bruker-AXS) was applied for the quantitative analysis of the different Ni/Ti ratios. Fused beads (1 g sample and 8 g lithium tetraborate (≥99.9% Sigma-Aldrich) were prepared for all samples, using the XRS-XRFUSE6 (Bruker-AXS) melting system. The following instrument parameters were selected for the elemental analysis: Tube: Rh—anode 3 kW, voltage 50 kV current 60 mA, emission lines: Ni: Kα_1_ LiF(200), 7.5 keV, 48.67° *2θ*, Ti: Kα_1_, LiF(200), 86.12° *2θ*, detectors: proportional flow—and scintillation counter. For data evaluation the software SPECTRAplus in combination with the standard package GEO-QUANT ADVANCED (Bruker—AXS) was used.

### 2.3 Ex Situ and In Situ Powder X-ray Diffraction (PXRD)

Ex situ structural analysis of benchmark materials after selected treatments was carried out using a Rigaku SmartLab-SE instrument with Bragg–Brentano geometry (Co–Kα_1/2_, λ = 1.7890 Å) using a D/teX Ultra 250 compound silicon strip 1D-detector (Rigaku, Tokyo, Japan). The ground samples were placed on a glass-sample holder with patterns recorded between 10° to 90° *2θ* with a step size of 0.01°.

In situ analysis of LaNiO_3_ in 10% H_2_/He up to 800 °C was carried out using a Rigaku SmartLab instrument (Cu–Kα_1/2_, λ = 1.54059 Å) using a HyPix-3000 detector in 1D scan mode with a Reactor X high-temperature attachment. The powder was placed on a silica sample holder with patterns recorded in a range of 15° to 70° *2θ* with a step size of 0.01°.

Rietveld refinement with a full axial model was done using the program TOPAS 7.0.^23^ A Double-Voigt approach was applied to calculate the crystallite sizes. The resolution function of the diffractometers was obtained from the structure refinement of a LaB_6_ standard.

### 2.4 Scanning Electron Microscopy (SEM)

Scanning electron microscopy and corresponding energy-dispersive X-ray spectroscopy (EDX) measurements were performed on a field-free analytical Tescan Clara ultrahigh-resolution scanning electron microscope operated at 25 kV. EDX maps were collected using an Oxford Ultim Max 60 mm^2^ detector.

### 2.5 Catalytic Dry Reforming Measurements (DRM)

200 mg of sample powder was fixed with silica wool in a home-built 7 mm (inner diameter) silica glas tube flow reactor setup under a total continuous gas flow of 60 mL min^–1^ (CH_4_:CO_2_:He = 1:1:1 mL min^–1^). For the DRM experiments, the reactor was heated with 5 °C min^–1^ to 800 °C, followed by an isothermal period at 800 °C for 30 min. For H_2_ treatments the reactor was heated with 50 °C min^–1^ to 1000 °C, followed by an isothermal period for 30 min with a gas flow of 20 mL min^–1^ H_2_. The gas flow of all used gases was controlled by mass flow controllers (MKS Instruments).

An external S-type thermocouple was placed in close contact to the reactor tube to ensure the correct temperature reading. Moreover, an independent calibration of a potential temperature gradient between the external thermocouple and the exact location of the catalyst bed was performed by inserting a second thermocouple into the gas flow inside the reactor tube. The output gas was directly detected by an online quadrupole mass spectrometer (Balzers QME 125). CO_2_, CH_4_, CO and H_2_ were measured at their respective *m*/*z* ratios of *m*/*z*= 44, 16, 28 and 2, respectively. Relevant fragmentation patterns have been considered as well. For the display of the catalytic data, we show the conversion of the relevant signals as a qualitative measure of the catalytic activity, as due to the ongoing structural transformations during the DRM reaction, active-site-normalized reaction rates (and consequently, TOF values) cannot be reliably calculated. The conversion for each gas phase is calculated as a fraction of the mass spectrometer (MS) ion current [E^–10^A] divided by the MS signal at room temperature after calibrating the flow rate of each gas phase. The measured He signal was used to correct fluctuations in the MS signals. For CO_2_ the calculation is as follows:

Conversion[%]=(1−CO2[E−10A]He[E−10A]CO2initial[E−10A]Heinitial[E−10A])×100

Since the CO_2_ conversion is indicative of CO, and CH_4_ conversion of H_2_ production, the H_2_/CO ratio was calculated from these conversions, respectively.

### 2.6 Temperature-Programmed Hydrogen Reduction (TPR) and Desorption (TPD)

Hydrogen temperature-programmed reduction (H_2_-TPR) and temperature-programmed desorption (TPD) measurements were performed in a setup for volumetric adsorption of gases consisting of a silica tubular reactor (V = 36 mL). H_2_-TPR is conducted under static conditions. As for H_2_-TPR, a defined amount of about 70 mg of the powder sample was fixed by silica wool in the reactor in a chemically inert environment. Catalysts are pretreated in flowing dry O_2_ for 1 h at 700 °C and subsequently cooled down in O_2_ atmosphere. In order to desorb surface- and physically adsorbed oxygen, samples are evacuated up to a defined base pressure of 10^–6^ mbar. During H_2_-TPR, a defined (ca. 490 mbar) amount of predried H_2_ (using a liquid N_2_ cooling trap) was expanded from a separately calibrated volume (9 mL) into the entire reactor volume. A zeolite trap is installed after the reactor to remove reaction-formed water.

After equilibration of the final reactor pressure, the temperature program was started to measure the H_2_ uptake up to 900 °C. The temperature program included a heating phase from 25 to 900 °C at a rate of 10 °C min^–1^, followed by an isothermal period at 900 °C for 10 min, and finally, a cooling process to room temperature (rate 10 °C min^–1^). Additional information, including the technical information on the setup, test conditions, and method of calculation of the H_2_-uptake, is provided in ref.^21^ A QMS system from Balzers (QMA 125; QME 125–9) was employed for continuous gas analysis during TPD measurements. Relevant gases were detected by their respective molecular ions: H_2_ (*m*/*z* 2), H_2_O (*m*/*z* 17/18), CO (*m*/*z* 28), O_2_ (*m*/*z* 32), CO_2_ (*m*/*z* 44). After the H_2_-TPR phase, the samples are evacuated at 25 °C to 10^–6^ mbar and subsequently, heating was started with the same temperature program as for the H_2_-TPR tests. The only dominant desorbed species in all TPD tests was H_2_. The H_2_ MS signal was calibrated via a separate effusion experiment, where H_2_ was pumped from the reactor (without sample) via a capillary leak into the QMS chamber. This way, the effusion rate – calculated from absolute pressure decrease vs time in the reactor chamber – was obtained in [mol s^–1^] and correlated to the respective H_2_ MS signal. Based on this calibration function, the H_2_ desorption rates were calculated from the TPD MS signal.

### 2.7 BET and Metal Dispersion Measurements

Both BET specific surface areas and metal dispersion of Ni were measured on an Anton Paar Autosorb 6300 PFE XR. Nitrogen was used as adsorbent at −196 °C for the BET measurements after degassing the samples *in vacuo* at 200 °C for 1 h (Figure S1). For calculating the Ni dispersion, static volumetric H_2_ chemisorption experiments were carried out at 40 °C. To induce Ni exsolution, the samples were uniformly reduced in H_2_ at 1000 °C for 30 min, followed by cooling *in vacuo* to remove any excess H_2_. The final calculations were based on a Langmuir fit of the measured isotherms. The respective surface areas and dispersion are discussed in Section 3.1.

### 2.8 In Situ Thermogravimetry (TGA) and Differential Thermal Analysis (DTA)

Thermogravimetric analysis (TGA) and associated differential thermal analysis (DTA) have been carried out on a Netzsch STA Jupiter 449 F1 apparatus using a SiC furnace up to 1600 °C and corundum sample holders. For in situ analysis during hydrogen reduction, measurements were conducted from 25 °C – 800 °C at 10 °C min^–1^ with a gas flow of 180 mL min^–1^ Argon and 20 mL min^–1^ H_2_ at ambient pressure. The system is coupled to a Pfeiffer Prisma quadrupole mass spectrometer for simultaneous gas phase analysis.

### 2.9 X-ray Photoelectron Spectroscopy (XPS)

Surface chemical analysis was carried out on a Thermo Fisher X-ray spectrometer with a Mg/Al dual anode. For all measurements, an Al–Kα X-ray source was used. Perovskite powders were fixed on stainless steel holders using conductive carbon tape. Qualitative analysis was based on the Ni 2p, La 3d, Ti 2p, O 1s and C 1s high-resolution spectra. Chemical shifts were internally calibrated to the La 3d_5/2_ component. Fitting of the spectra by different components and oxidation states was performed using literature-reported constraints for the full-width-at-half-maximum and the binding energies, where applicable. Background correction was done using Shirley- and Tougaard-type functions.

## 3 Results and Discussion

### 3.1 Influence of Ti Doping on the Structure and Morphology of LaNiO_3_ Perovskite DRM Catalysts

The target phases for the catalyst precursors are selected compositions with representative and fixed Ti doping levels in LaNiO_3_ within the full compositional perovskite series LaNi_*x*_Ti_1–*x*_O_3_. Composition-wise, XRF measurements to verify the nominal composition from synthesis are in good accordance with the experimentally targeted nominal compositions (Table 1). The maximum deviation of x is ± 0.03.

**Table 1:** Nominal and Experimentally Determined XRF Compositions of All Studied LaNi_*x*_Ti_1–*x*_O_3_ Compositions

x_target_	Ni/Ti_target_	Ni/Ti_measured_	x_calculated_
0.75	3.00	3.51	0.78
0.50	1.00	0.90	0.47
0.25	0.33	0.38	0.27
0.20	0.25	0.24	0.19
0.15	0.18	0.18	0.15
0.10	0.11	0.11	0.10
0.05	0.05	0.05	0.05

Doped La­(Ni,Ti)­O_3_ generally exhibits a perovskite type structure with the trigonal space group *R*3̅*c*. With higher Ti content in the structure (x_Ni_ < 0.80) the symmetry changes to the orthorhombic space group *Pbnm*.^24^ At the Ni-free La_2_O_3_/TiO_2_ end of the La_2_O_3_/NiO/TiO_2_ compositional series is pyrochlore-type La_2_Ti_2_O_7_ with a monoclinic *P*112_1_ symmetry. The maximum Ni/Ti ratio at which the perovskite changes the symmetry and the pyrochlore phase is formed, is hitherto unknown. Figure 1 shows a representative VESTA-drawn scheme of a doped La­(Ni,Ti)­O_3_ material (Panel **a**) in comparison with La_2_Ti_2_O_7_ (Panel **b**).

In the as-calcined state for compositions between 0.20 < x_Ni_ < 1.00 phase-pure perovskites with crystallite sizes between 10 to 40 nm, as determined by Rietveld refinement, are observed by PXRD (Figure 2 and Table 2). Higher Ti doping levels cause the formation of progressively increasing amounts of La_2_Ti_2_O_7_, reaching a weight fraction of 80 wt % at x_Ni_ = 0.05 (Table 2). An attempt to determine the La_2_Ti_2_O_7_ formation threshold was made via electron beam microprobe using wavelength-dispersive X-ray spectroscopy (WDX). However, after 21 days of sintering the crystallites in the sample were still not large enough to get any accurate measurements. In essence, the lowest amount of Ni that can be tolerated within the perovskite lattice is found in the range between 0.25 ≥ x_Ni_ ≥ 0.20. Restricted to the compositional range, where only La­(Ni,Ti)­O_3_ is stable, the cell parameters of La­(Ni,Ti)­O_3_ increase if more Ti is added (Table 3). This effect can be explained by the larger ionic radii of the B-site ions introduced upon doping. In pure LaNiO_3_ only ^VI^Ni^3+^ with a radius of 0.56 Å – 0.6 Å^26^ is found on the B-site. Upon introduction of Ti, the B-site is occupied by both ^VI^Ni^2+^ and ^VI^Ti^4+^ with respective radii of 0.69 Å and 0.61 Å^26^. As the amount of Ni is further reduced, Ti has to compensate and also form either ^VI^Ti^3+^ or ^VI^Ti^2+^ with radii of 0.67 Å and 0.86 Å^26^ respectively, counteracting the increase of the unit cell parameters.

**Figure 2:**
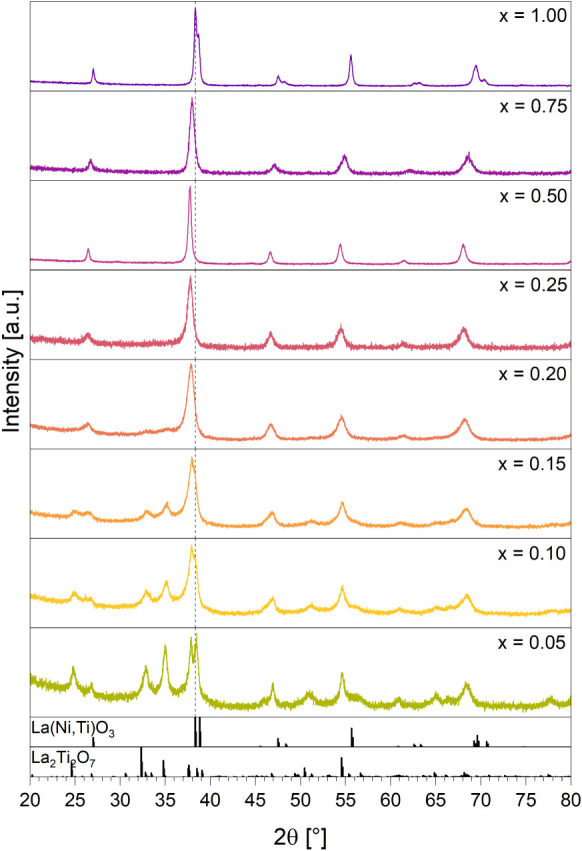
PXRD patterns of the as calcined LaNi_*x*_Ti_1–*x*_O_3_ compositional series. The dotted line at 38.3° *2θ* serves to highlight the shift of the main La­(Ni,Ti)­O_3_ peak due to changes in cell parameters with increased Ti content (cf. Table 3). References: La­(Ni,Ti)­O_3_ modified after^25^ and,^27^ La_2_Ti_2_O_7_.^26^

**Table 2:** Calculated Compositions and Crystallite Sizes of as Calcined LaNi_*x*_Ti_1–*x*_O_3_ Samples^a^

LaNi_x_Ti_1‑x_O_3_ Sample	Phase	Weight Fraction [wt %]	CrySize [nm]
x_Ni_ = 1.00	LaNiO_3_	100	40
x_Ni_ = 0.75	La(Ni,Ti)O_3_	100	25
x_Ni_ = 0.50	La(Ni,Ti)O_3_	100	30
x_Ni_ = 0.25	La(Ni,Ti)O_3_	100	20
x_Ni_ = 0.20	La(Ni,Ti)O_3_	60	15
	La_2_Ti_2_O_7_	40	10
x_Ni_ = 0.15	La(Ni,Ti)O_3_	60	10
	La_2_Ti_2_O_7_	40	10
x_Ni_ = 0.10	La(Ni,Ti)O_3_	30	15
	La_2_Ti_2_O_7_	70	10
x_Ni_ = 0.05	La(Ni,Ti)O_3_	20	25
	La_2_Ti_2_O_7_	80	20

a Numbers in Column 3 refer to the weight percentages of the phases obtained from a quantitative phase analysis based on the Rietveld refinement. The accuracy of all weight fractions is estimated to be ± 1–3 wt.%.

**Table 3:** Lattice Parameters of the LaNi_*x*_Ti_1–*x*_O_3_ Perovskite Solid Solution (x_Ni_ = 1.00, 0.75, 0.50 and 0.25)^a^

Sample	a [Å]	b [Å]	c [Å]	V [Å^3^]	Space Group
x_Ni_ = 1.00	5.46	-	13.19	340.3	*R*3̅*c*
x_Ni_ = 0.75	5.47	5.52	7.81	236.0	*Pbnm*
x_Ni_ = 0.50	5.52	5.56	7.86	241.0	*Pbnm*
x_Ni_ = 0.25	5.51	5.54	7.87	240.4	*Pbnm*

a Due to overlapping peak positions of La­(Ni,Ti)­O_3_ and La_2_Ti_2_O_7_, the cell parameters of the remaining perovskite cannot be calculated accurately when La_2_Ti_2_O_7_ is present.

We have further characterized the structure and morphology by combined SEM/EDX (Figure S2) to assess the influence of Ti doping. As for the overall morphology, the influence of both progressive Ti doping and formation of increased amounts of La_2_Ti_2_O_7_ is minute. Micrometer-sized irregular grains are observed for all samples, including the respective Ni-free and Ti-free end compositions. The only marked difference is the appearance of a grid-like pattern of interconnected grains with a porous structure for the respective Ni-rich compositions x_Ni_ = 1.00, 0.75, and 0.50. This structure features a porous arrangement of irregular holes of about 100 nm and for the Ti-doped samples, and as judged by the respective EDX patterns (shown for x_Ni_ = 0.50), do not exhibit any chemical inhomogeneity. Also at micrometer-scale, no such chemical heterogeneity is observed for any of the samples.

To gain insight into the specific surface area and respective porosity of the Ti-doped samples, we have accordingly measured full sets of nitrogen adsorption–desorption isotherms at −196 °C. The isotherms are shown in Figure S1, the DFT-fitted cumulative pore volume as a function of pore radius in Figure 3.

**Figure 3:**
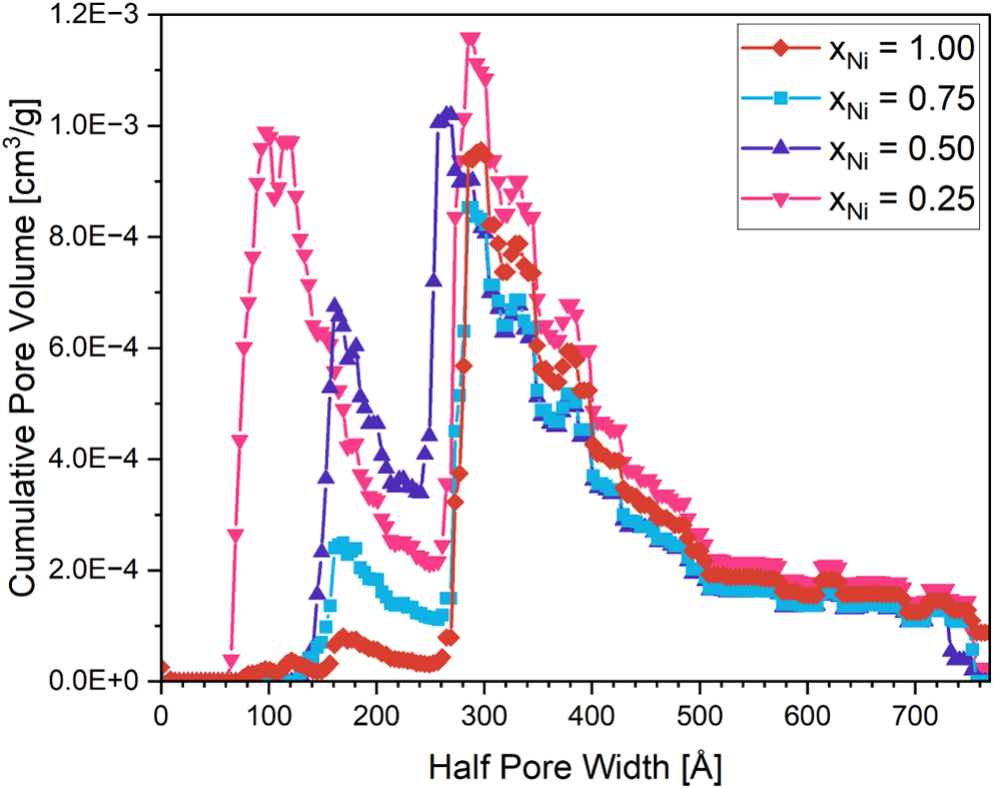
DFT-fitted cumulative pore volume as a function of pore radius for the LaNi_*x*_Ti_1–*x*_O_3_ samples with x_Ni_ = 1.00, 0.75, 0.50 and 0.25.

The respective BET surface areas have been determined as 4.6 m^2^ g^–1^, 4.3 m^2^g^–1^, 5.9 m^2^ g^–1^, and 12.1 m^2^g^–1^ for x_Ni_ = 1.00, 0.75, 0.50, and 0.25. We have restricted this analysis to the samples where no La_2_Ti_2_O_7_ is present to avoid influences of a second phase. A clear trend in the surface area upon Ti incorporation is evident: the higher the Ti amount, the higher the specific surface area, which resembles results obtained for Mn-substituted La–Ni perovskites.^28^ The same trend is reflected in the cumulative pore volume (Figure 3). LaNiO_3_ without Ti features a distinct pore volume peak with a pore radius of around 30 nm and a smaller peak for pores with a radius of around 18 nm. The pores with larger radius are seemingly invariant of Ti doping down to x_Ni_ = 0.25. In contrast, upon Ti doping, the smaller pores get increasingly more pronounced and almost equals the peak at 30 nm pore radius in intensity. Interestingly, this peak shifts to a pore radius of ca. 10 nm for x_Ni_ = 0.25, although both XRD and SEM do not show significant changes in structure or morphology. Ti doping apparently also increases the cumulative total pore volume and also leads to a broader pore size distribution, at least for x_Ni_ = 0.25.

Ti doping appears to have no major impact on the metal dispersion after H_2_ treatment of the perovskites (Table 4). The presence of Ti reduces the metal dispersion only to a very minor degree when compared to samples without Ti. However, increasing amounts of Ti do not further reduce the Ni dispersion as all Ti containing samples essentially show the same degree of dispersion.

**Table 4:** H_2_ Monolayer Uptake at 40 °C and the Calculated Metal Dispersion of the LaNi_*x*_Ti_1–*x*_O_3_ Perovskite Solid Solutions (x_Ni_ = 1.00, 0.75, 0.50 and 0.25) Following Reduction at 1000 °C for 30 Min

Sample	H_2_ uptake [μMol/g]	Ni dispersion [%]
x_Ni_ = 1.00	26.50	1.26
x_Ni_ = 0.75	16.29	1.04
x_Ni_ = 0.50	10.76	1.03
x_Ni_ = 0.25	5.26	1.02

### 3.2 Influence of Ti Doping on the Stability and Ni Exsolution Behavior under Hydrogen and DRM Atmospheres

As Ni exsolution from perovskites usually is a prerequisite to prepare highly active perovskite-based DRM catalysts, we have accordingly assessed the general structure- and phase stability of all Ti-doped samples in hydrogen and under DRM conditions. The difference between the two treatments is the reduction strength of the gas atmosphere. For Ti-free LaNiO_3_, we have already assessed the respective differences in the decomposition process and the appearance of intermediate phases. LaNiO_3_ can be decomposed in hydrogen fully to Ni and La_2_O_3_, without the intermediate Ruddlesden–Popper La_2_NiO_4_ phase.^6^ However, LaNiO_3_ can also be self-activated in the DRM mixture without hydrogen pretreatment. The reduced reduction strength of the DRM atmosphere (hydrogen is only accumulated as a reaction product at advanced stages of DRM operation) intermediarily stabilizes the La_2_NiO_4_ phase so that it can be observed in PXRD patterns during DRM.^6^ Figure 4 shows that all samples decompose in hydrogen, but with increasing Ti content higher amounts of the perovskite precursor remain after similar hydrogen treatments (Table 5). The hydrogen-treated samples can be divided into three categories: (1) x_Ni_ = 1.00 and 0.75, where La_2_O_3_ is present after reduction, (2) x_Ni_ = 0.50 and 0.25 where La_2_TiO_5_ is formed and (3) x_Ni_ < 0.25, where Ni is exsolved, but both precursor phases remain without La_2_TiO_5_ formation. Several trends in the stability with pronounced impact on the DRM performance are evident:

(1) In general, Ti doping of LaNiO_3_ enhances the stability of the perovskite precursor and La_2_TiO_5_ acts as a Ti sink at low Ti doping levels.
(2) La_2_O_3_ in different modifications is only formed for Ti-free LaNiO_3_ and very low Ti doping levels of x_Ni_ = 0.75. The dominant La_2_O_3_ polymorph is trigonal (space group *P*3̅*m*1) with minor contributions of two cubic modifications (space groups*Ia*3̅ and *Im*3̅*m*, respectively). For x_Ni_ = 0.50 and higher Ti levels, all La available through partial perovskite precursor decomposition is bound as La_2_TiO_5_ and below x_Ni_ = 0.25, as a La_2_Ti_2_O_7_ pyrochlore phase. As will be discussed below, this suppression of La_2_O_3_ formation potentially causes a switch in the DRM reaction mechanism from a La-oxycarbonate one at lower Ti doping levels to a vacancy-dominated one at progressively higher Ti doping levels.
(3) If we calculate the nominal Ni amount and compare it to the amount of exsolved Ni determined by Rietveld refinement, we conclude that these amounts match within the measurement uncertainty and, thus, all nominally available Ni is leached from the perovskite during hydrogen reduction. The remaining perovskite, hence, approximately has a composition of LaTiO_3_ and no substantial influence of Ti doping on the Ni exsolution capability is evident.
(4) The amount of La_2_TiO_5_ decreases from x_Ni_ = 0.75 to x_Ni_ = 0.25 and accordingly, the amount of the remaining perovskite increases, reflecting the stabilization ability of Ti.
(5) At and below x_Ni_ = 0.20 the only three phases that are present are La_2_Ti_2_O_7_, LaTiO_3_ and metallic Ni in varying compositions. La_2_Ti_2_O_7_ appears at first at x_Ni_ = 0.20 and its weight fraction increases from 44 wt % to 87 wt % at x_Ni_ = 0.05. The amount of perovskite accordingly decreases. As the determination of the very low amounts of Ni is challenging for x_Ni_ = 0.05, Figure S3 shows the corresponding Rietveld refinement fit.
(6) Due to the stabilizing nature of Ti, self-activation in terms of perovskite decomposition and Ni exsolution of LaNi_*x*_Ti_1–*x*_O_3_ under DRM conditions is only possible for x_Ni_ = 1.00, x_Ni_ = 0.75 and x_Ni_ = 0.50 (Figure 5). The marker for self-activation of those samples is the appearance of La_2_O_3_, monoclinic La_2_O_2_CO_3_ (for the former two) and metallic Ni (for all samples). For x_Ni_ = 0.50, the perovskite structure remains stable, and no La_2_O_3_ is observed despite Ni exsolution. Three different compositional boundaries with respect to self-activation therefore exist: (1) between x_Ni_ = 1.00 and x_Ni_ = 0.75, total or partial decomposition of the perovskite phase is observed, accompanied by Ni exsolution and formation of La_2_O_3_ and monoclinic La_2_O_2_CO_3_; (2) between x_Ni_ = 0.75 and x_Ni_ = 0.50, Ni exsolution is observed, but no perovskite decomposition; (3) for x_Ni_ < 0.50, the perovskite persists and no exsolution is observed. Correlated to the DRM performance, activity is observed down to x_Ni_ = 0.50, but for compositions x_Ni_ < 0.50, also no activity evolves. The exclusive Ni exsolution without substantial effect on the perovskite structure for x_Ni_ = 0.50 is also evident from the steep activity increase at *T* = 750 °C, which is directly associated with the appearance of metallic Ni and the reduction of NiO in the DRM reaction mixture. For x_Ni_ ≥ 0.75, processes before the final decomposition of the perovskites (e.g., transient formation of La_2_NiO_4_ or La_2_O_2_CO_3_ phases) give rise to intermediate acceleration of DRM activity. An according Rietveld refinement fit for La_2_O_2_CO_3_ is also shown in Figure S3.

**Figure 4:**
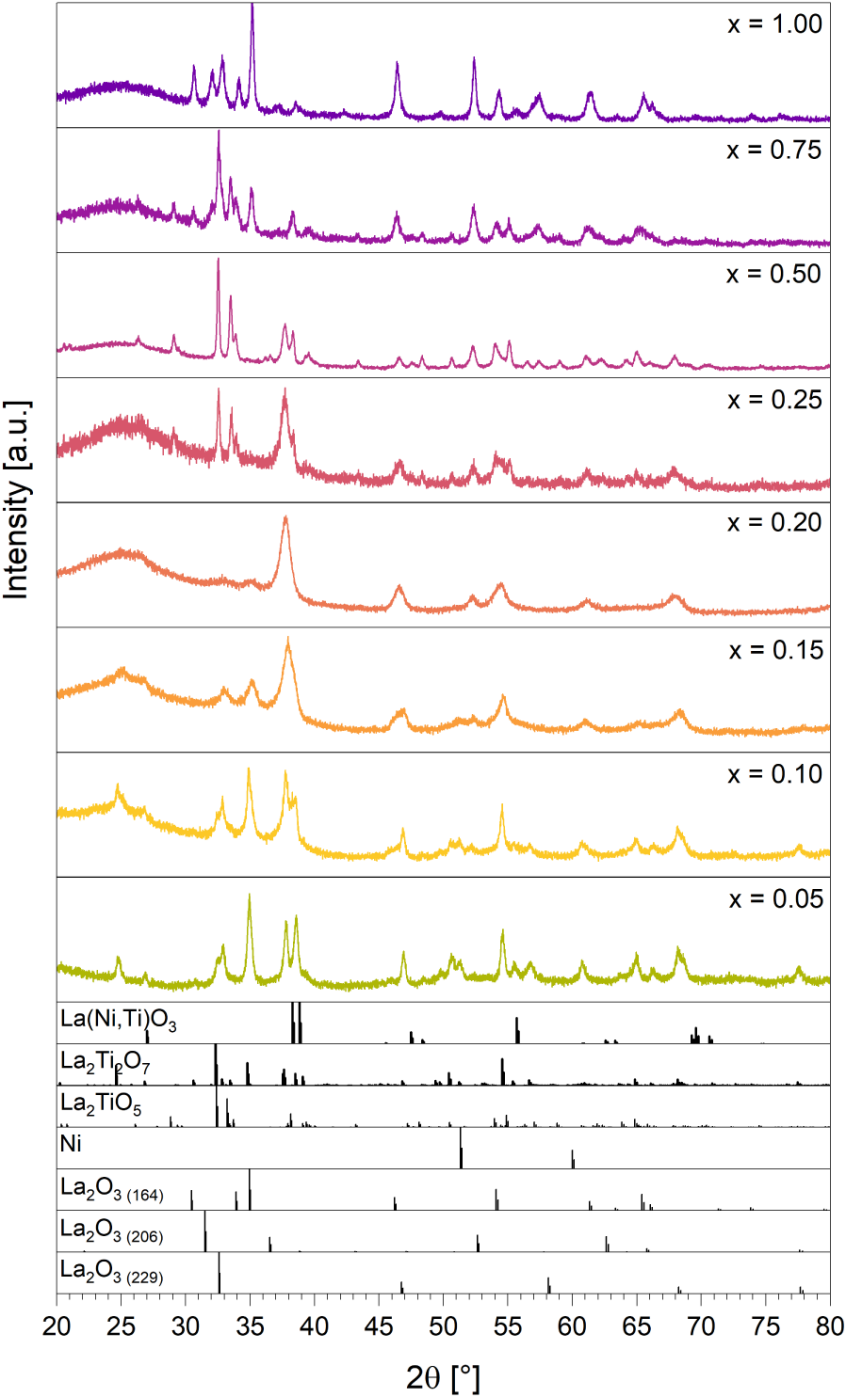
PXRD results of the LaNi_*x*_Ti_1–*x*_O_3_ samples after treatment in hydrogen at 1000 °C for 30 min. For the specific compositions of each sample we refer to Table 5. References: La­(Ni,Ti)­O_3_ modified after^25^ and,^27^ La_2_Ti_2_O_7_,^26^ La_2_TiO_5_,^29^ Ni,^30^ La_2_O_3 (164)_,^31^ La_2_O_3 (206)_,^32^ La_2_O_3 (229)_.^33^

**Table 5:** Calculated Compositions, Phases and Crystallite Sizes of the LaNi_*x*_Ti_1–*x*_O_3_ Samples After Treatment in 1 Bar H_2_ at 1000 °C for 30 Min Derived from Quantitative Rietveld Phase Analysis^a^

LaNi_x_Ti_1‑x_O_3_ Sample	Phase	Weight Fraction [wt.%]	CrySize [nm]
x_Ni_ = 1.00	La_2_O_3 (164)_	29	50
	Ni	60	60
	La_2_O_3 (229)_	10	30
	La_2_O_3 (206)_	1	25
x_Ni_ = 0.75	La_2_TiO_5_	72	40
	Ni	20	45
	La_2_O_3 (164)_	7	35
	La_2_O_3 (206)_	1	-
x_Ni_ = 0.50	La_2_TiO_5_	58	65
	Ni	13	35
	LaTiO_3_	28	25
	La_2_NiO_4_	1	-
x_Ni_ = 0.25	LaTiO_3_	62	15
	Ni	10	30
	La_2_TiO_5_	28	50
x_Ni_ = 0.20	LaTiO_3_	50	15
	Ni	6	30
	La_2_Ti_2_O_7_	44	10
x_Ni_ = 0.15	La_2_Ti_2_O_7_	66	10
	Ni	3	-
	LaTiO_3_	31	15
x_Ni_ = 0.10	La_2_Ti_2_O_7_	77	20
	Ni	4	20
	LaTiO_3_	19	15
x_Ni_ = 0.05	La_2_Ti_2_O_7_	87	35
	Ni	1	-
	LaTiO_3_	12	30

a The accuracy of all weight fractions is estimated to be ± 1–3 Wt.%.

**Figure 5:**
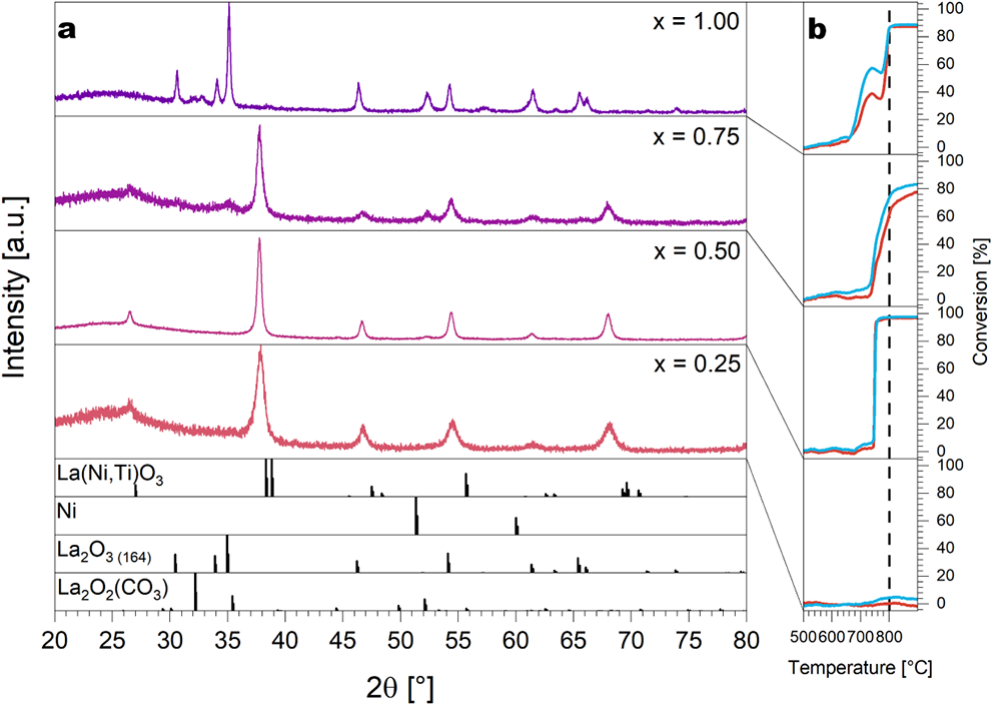
Panel **a** - PXRD patterns of the LaNi_*x*_Ti_1–*x*_O_3_ samples after treatment in the DRM mixture without prior hydrogen treatment. Panel **b** - corresponding DRM profiles. Blue traces refer to CO_2_, red traces to CH_4_ conversion. Heating rate: 5 °C min^–1^, sample mass: 200 mg each, gas flow: CO_2_:CH_4_:He = 20 mL:20 mL:20 mL, GHSV: 18 Lh^1–^g_cat_^–1^.

In order to back up the ex situ PXRD stability tests, we further provide in situ thermogravimetry/differential thermoanalysis and additional in situ PXRD data for the representative LaNiO_3_ material during hydrogen reduction (Figure 6). Generally, the observed mass loss in the TG signal during treatment in a 10% H_2_/Ar mixture matches the increasing stability trend with increasing Ti content (Figure 6, Panel a). The mass loss is most pronounced for LaNiO_3_, where it exactly matches the one expected for oxygen removal from the perovskite structure during full transformation of LaNiO_3_ to Ni and La_2_O_3_ according to the overall decomposition reaction 2LaNiO_3_ → 2Ni + La_2_O_3_ + 1.5O_*2*_. Also pronounced are the respective mass losses for x_Ni_ = 0.75 and x_Ni_ = 0.50, while for x_Ni_ < 0.50, hardly any loss is observed. As highlighted in Figure 6, Panel b, these differences are also reflected in the respective differentiated DTA (DDTA) signals. For LaNiO_3_, these data are directly related to the associated in situ PXRD diffractograms (Figure 6c), obtained under exactly the same experimental conditions. We note, that mass loss, DDTA signals and the changes in the PXRD patterns exactly coincide temperature-wise.

**Figure 6:**
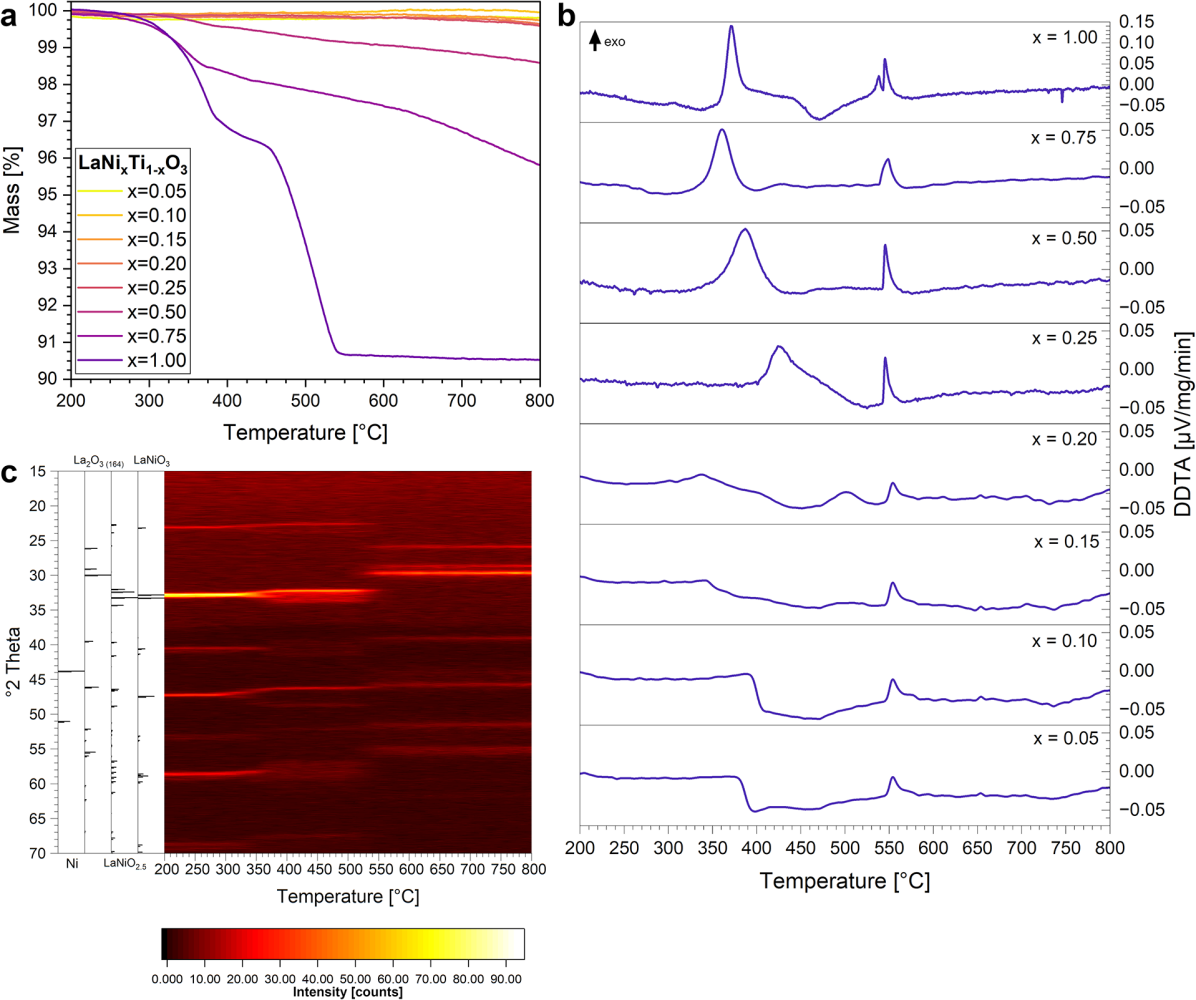
Panel **a** - Mass loss of the LaNi_*x*_Ti_1–*x*_O_3_ samples from 200 °C – 800 °C in 10% H_2_/Ar. Panel **b** - Differentiated DTA signals of LaNi_*x*_Ti_1–*x*_O_3_ samples from 200 °C – 800 °C in 10% H_2_/Ar. Panel **c** - In situ PXRD of LaNiO_3_ from 200 °C – 800 °C in 10% H_2_/He. A heating rate of 10 °C min^–1^ was used for both PXRD and TG/DTA measurements.

For LaNiO_3_, two sharp endothermal signals are observed, which almost exactly match the inflection points of the two-step mass loss. In combination with PXRD (Figure 6, Panel c) we are able to relate the first pronounced peak to the phase transformation LaNiO_3_ → LaNiO_2.5_ + 0.25O_2_ (according to0.5O) (Step 1) and the second one to the transformation 2LaNiO_2.5_ → 2Ni + La_2_O_3_ + O_2_(Step 2). With respect to the quantification of the mass loss, the ideal mass loss with respect to released oxygen observed during step 1 and step 2 amounts to 3.3% and 6.5%, in total 9.8% at 800 °C. The values for LaNiO_3_ are very close to these values (3.5% and 6.0%, in total 9.5% at 800 °C), indicating full decomposition. The respective mass losses for the samples with x_Ni_ = 0.75, 0.5, and 0.25 are consequently much lower (x_Ni_ = 0.75:1.8% and 2.3%, in total 4.1%; x_Ni_ = 0.50:0.5% and 0.8%, in total 1.3%; x_Ni_ = 0.25:0.1% and 0.3%, in total 0.4%), further emphasizing the stabilization role of Ti, which gets more pronounced up to x_Ni_ = 0.25 before the formation of La_2_Ti_2_O_7_ sets in. Both steps are also reflected in the respective DDTA peaks (Figure 6, Panel b) and the associated structural changes in the in situ PXRD peaks (Figure 6, Panel c). In situ PXRD reveals two pronounced changes in the phase composition: the transformation from LaNiO_3_ to LaNiO_2.5_ between 330 and 340 °C, and then Ni exolution and decomposition of the perovskite precursor starting between 525 to 540 °C. At more or less exactly the same temperatures, the DDTA signal reveals two pronounced peaks, which can now be directly associated to the two transformation steps discussed above. The one at higher temperatures is present for all Ti-doped samples and refers to Ni exsolution, as it is observed for all samples irrespective of Ti doping. The first DDTA peak is then associated with formation of LaNiO_2.5_ from LaNiO_3_ by oxygen removal. It is very pronounced for x_Ni_ = 1.00, 0.75, 0.5 and 0.25, but severely broadened and less distinguishable for x_Ni_ ≤ 0.20. As deduced from PXRD, this is most likely due to the beginning presence of La_2_Ti_2_O_7_. For x_Ni_ ≤ 0.10, the first peak is hardly discernible anymore.

With respect to a different reduction and exsolution behavior, we have also examined the catalysts by SEM/EDX after hydrogen reduction (Figure S4). Ni exsolution is verified for all samples from x_Ni_ = 1.00 to 0.20, as evidenced by line profiles over several exsolved small Ni particles (line profiles shown in yellow, some of those Ni particles have been marked by yellow arrows). For x_Ni_ ≤ 0.15, we could not resolve the small Ni particles.

### 3.3 Influence of Ti Doping on the Reduction and Redox Properties: Static H_2_-Temperature-Programmed Reduction (H_2_-TPR) and Thermal Desorption (H_2_-TPD)

Complete decomposition or partial reduction of Ni-containing perovskites under DRM reaction conditions—leading to the formation of metallic nickel particles—can significantly affect the reaction kinetics.^6,15^ In addition to the role of exsolved metallic nickel, the presence of oxygen vacancies on the surface and its impact on CO_2_ activation has been previously reported.^34^ Therefore, it is expected that even slight surface reduction of structurally stable samples under reaction conditions could be effective in improving the reactivity. Thus, the reducibility of the catalysts in hydrogen has been evaluated under static conditions and the results are summarized in Figure 7, as well as in Figure S5, including quantification in Table 6.

**Figure 7:**
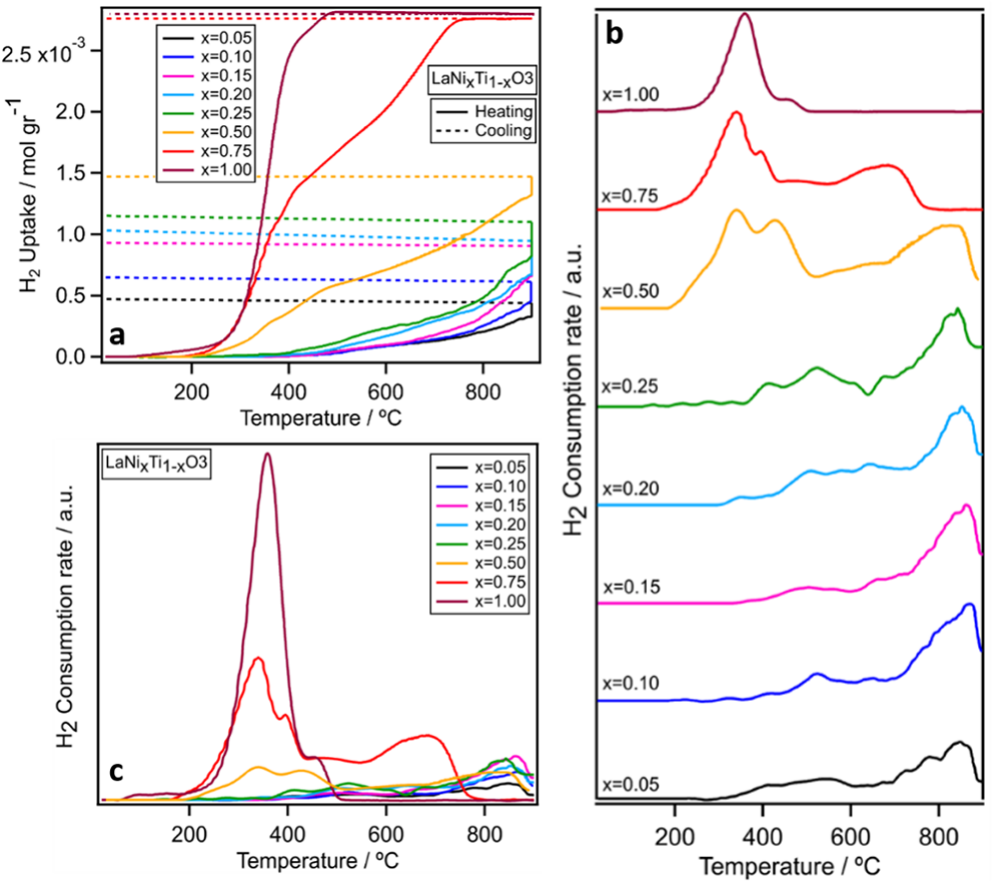
Panel **a** - Integral H_2_ uptake during static temperature-programmed H_2_ reduction on LaNi_*x*_Ti_1–*x*_O_3_, x_Ni_ = 0.05 to 1.00 after preoxidation in flowing O_2_ using an initial H_2_ pressure of 125 mbar. Temperature program: heating from 25 to 900 °C (10 °C min^–1^/full lines), followed by an isothermal period at the maximum temperature (900 °C for 10 min/full line) and a cooling phase (10 °C min^–1^/dashed line). Panel **b** - Temperature-dependent first derivative of the H_2_ uptake during static temperature-programmed H_2_-reduction on LaNi_*x*_Ti_1–*x*_O_3_, x_Ni_ = 0.05 – 1.00 catalysts recalculated according to the data from Panel A. Panel **c** is plotted based on the same data as Panel B for direct intensity comparison.

**Table 6:** Amounts of the Integral H_2_ Uptake, Bound Hydrogen (H_2_ Desorption) and Number of Reduced Sites during TPR

Sample	Integral H_2_ Uptake in H_2_-TPR/mol g^–1^	Integral H_2_ desorption in TPD/mol g^–1^	Number of reduced sites in TPR/mol g^–1^
x_Ni_ = 0.05	4.72 · 10^–4^	0.79 · 10^–4^	3.93 · 10^–4^
x_Ni_ = 0.10	6.47 · 10^–4^	0.67 · 10^–4^	5.80 · 10^–4^
x_Ni_ = 0.15	9.30 · 10^–4^	1.05 · 10^–4^	8.25 · 10^–4^
x_Ni_ = 0.20	1.03 · 10^–3^	0.77 · 10^–4^	9.53 · 10^–4^
x_Ni_ = 0.25	1.15 · 10^–3^	0.65 · 10^–4^	1.09 · 10^–3^
x_Ni_ = 0.50	1.5 · 10^–3^	2.74 · 10^–4^	1.23 · 10^–3^
x_Ni_ = 0.75	2.76 · 10^–3^	0.97 · 10^–4^	2.63 · 10^–3^
x_Ni_ = 1.00	2.80 · 10^–3^	0.53 · 10^–4^	2.75 · 10^–3^

The solid lines in Figure 7, Panel a show the integral H_2_ uptake profiles on LaNi_*x*_Ti_1–*x*_O_3_ (x_Ni_ = 0.05–1.00) during static H_2_-TPR from 25 °C up to 900 °C, as well as during the isothermal phase at 900 °C for 10 min. In order to more easily identify consumption peaks, the temperature- dependent first derivative of the integral H_2_ uptake, i.e., the uptake rate, is additionally shown in Figure 7, Panel b. The major detectable H_2_ uptake for x_Ni_ = 0.05 to 0.15 begins at temperatures higher than 450 °C. By decreasing the Ti doping level to x_Ni_ = 0.20 and 0.25 the reduction onset temperature shifts to around 400 °C and in the case of x_Ni_ = 0.50 and higher, the onset temperature significantly drops to around 200 °C. For x_Ni_ = 0.75 and x_Ni_ = 1.00 the appearance of the maximum of the main reduction peak around 350 °C (see Figure S5) in our study is in accordance with Ni-rich perovskites in literature.^8,35–38^ However, for these samples the reduction process stops at around 750 and 450 °C, respectively, because all hydrogen in the reactor is already consumed.

All the samples x_Ni_ ≤ 0.25 exhibit broad reduction peaks lower than 800 °C, which could be related to the reduction of near-surface sites (Figure 7). The distinguishable reduction peak at higher than 800 °C most likely is due to the already presence of exsolved tiny Ni particles.^15^ The integral H_2_ uptake increases steadily upon further heating and proceeds during the isothermal phase at 900 °C. Kinetic issues are most likely responsible for the ongoing H_2_ uptake during the isothermal phase.^39,40^ In general, the subsequent H_2_ consumption stagnates during the cooling phase. The dashed lines provide guides to the eye to show the difference between the integral H_2_ uptake at the end of the isothermal period and after cooling to room temperature. A minor increase of the H_2_ uptake is detected during the cooling phase, which most likely is due to adsorption/dissolution of H_2_ on/in the perovskite structure during temperature decrease and not to reduction. In order to quantify the amount of dissolved hydrogen during H_2_-TPR, additional temperature-programmed desorption (TPD) was conducted (Figure S5 together with quantitative results in Table 6). Additional information on the calculation procedure is available in ref.^15^ According to Figure S5, in general at least two distinguishable desorption peaks around 400 and 600 °C are present for all samples, which indicate different binding energies of hydrogen species.^41^ Quantitative results of Table 6 show that a small amount of the total hydrogen consumed during reduction is dissolved within the perovskite structure. In the case of x_Ni_ = 1.00 and 0.75, since all the hydrogen for H_2_-TPR is used up before the maximum temperature is reached, it is not possible to make a general judgment about the overall behavior of the catalysts in terms of the amount of dissolved hydrogen. However, this value is significantly higher for x_Ni_ = 0.50. As a conclusion for the redox properties we note that in addition to kinetical improvement of hydrogen reduction reaction at high Ni levels, there is a clear trend between final integral H_2_-uptake and Ni amount, which indicates that the reducible sites are mainly Ni sites.

We have roughly estimated the maximum amount of adsorption sites for H_2_ on LaNi_*x*_Ti_1–*x*_O_3_ (x_Ni_ = 1.00–0.50) using the BET surface area, as well as the molar mass and the density of the initial perovskites according to the following formula.^41,42^

$$N_{s,p}={\left(\frac{\rho .N_{A}}{M}\right)}^{2/3}\cdot N_{A}\cdot S_{s}\cdot m$$

*N*_s,p_ Total number of active sites of the perovskite

*ρ* Density of the perovskite/kg m^–3^

*M* Molar mass of the perovskite/kg mol^–1^

*N*_A_ Avogadro’s number; 6.022 × 10^23^ mol^–1^

*S*_s_ Specific surface area from BET/m^2^ kg^–1^

*m* Sample mass/kg

It was assumed that one H atom adsorbs on one surface unit cell and that the unit cells of all four initial perovskites exhibit the trigonal space group *R*3̅*c*. Based on this assumption, the final integral H_2_ uptake for x_Ni_ = 1.00 to 0.25 corresponds to about 670, 700, 240, and 103 monolayers H_2_, respectively, which indicates pronounced bulk reduction for all samples, corroborating the PXRD results. Note, that the amount of desorbed H_2_ during TPD after H_2_-TPR is much larger (at least 10 times more) than the monolayer capacity, which indicates dissolution of hydrogen within the perovskites structure, as discussed above.

### 3.4 Influence of Ti Doping on Methane Dry Reforming Performance and Catalyst Structure after DRM Operation

We have briefly assessed the self-activation of the LaNi_*x*_Ti_1–*x*_O_3_ samples in the DRM mixture in section 3.1. The present section now deals with the conversion profiles of the same samples after a uniform hydrogen prereduction step at 1000 °C to induce activation. In general, both the overall shape of the DRM profiles (Figure 8, Panel a) and the values of key parameters of catalytic performance (T_10_, T_50_ and T_90_ values, corresponding to 10%, 50% and 90% reactant conversion) strongly depend on the level of Ti doping. At first, we note that the onset temperature of catalytic activity shifts from about 320 °C for x_Ni_ = 1.00 and 0.75 to roughly 360 °C for x_Ni_ = 0.05.

**Figure 8:**
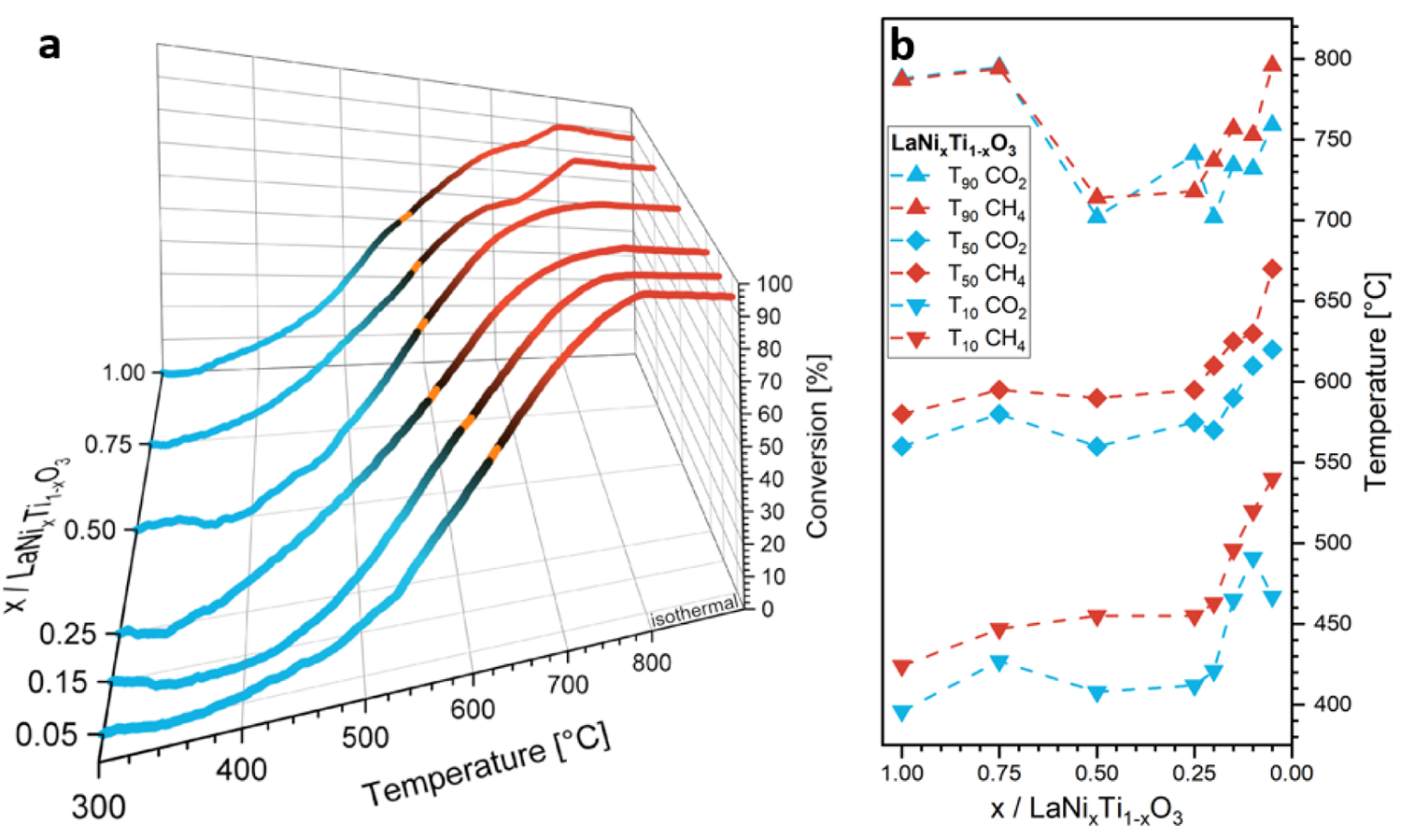
Panel **a** - CO_2_ conversion profiles of LaNi_*x*_Ti_1–*x*_O_3_ samples in DRM after reduction in H_2_ at 1000 °C for 30 min. Orange spots mark the T_50_ values. Samples with x_Ni_ = 0.20 and 0.10 are omitted for clarity. Panel **b** – Temperatures required for conversions of 10% (T_10_), 50% (T_50_) and 90% (T_90_). Heating rate: 5 °C min^–1^ from 50 °C – 800 °C followed by an isothermal period of 30 min, sample mass: 200 mg each, gas flow: CO_2_:CH_4_:He = 20 mL:20 mL:20 mL, GHSV: 18 Lh^1–^g_cat_^–1^.

Focusing on the catalytic conversion, displayed as T_10_, T_50_ and T_90_ values, a very strong response is found at compositions of x_Ni_ < 0.25. Matching the first appearance of La_2_Ti_2_O_7_, the formation of the latter is clearly detrimental to catalytic activity. For x_Ni_ < 0.25, the respective temperatures to reach all three conversion levels increases between 80 and 100 °C. However, based on the catalytic data we can conclude that the Ni content can be lowered to about a quarter (referenced to LaNiO_3_) to achieve the same catalytic turnover. Both perovskites with the lowest Ti content feature a worse T_90_ value than the samples with x_Ni_ = 0.50 and 0.25. The reason for this is evident in the respective DRM profiles (Figure 8, Panel a), which both feature a pronounced drop in activity at around 770 °C. This drop is absent in the profiles of all other samples. PXRD after DRM operation (Figure 9 and Table 5) and previous in situ PXRD data for LaNiO_3_ reveal for x_Ni_ = 1.00 and 0.75 the formation and presence of a La_2_O_2_CO_3_ phase.^6^ The decomposition of this phase releases CO_2_, and hence explains the drop in the CO_2_ conversion profiles. The cycled formation of this oxycarbonate phase has been suspected to be in the epicenter of DRM activity on LaNiO_3_^6^ and we hereby prove, that this mechanism still prevails at least down to x_Ni_ = 0.75. For x_Ni_ ≤ 0.50, as such an oxycarbonate phase is no longer observed, the DRM mechanism apparently switches to a different pathway. A comparable change in reaction mechanism was reported by Das et al.^43^ on A-site doped La_0.9_Sr_0.1_Ni_1–*x*_Fe_*x*_O_3_ catalysts in DRM. They report a La_2_O_2_CO_3_-mediated mechanism for La_0.9_Sr_0.1_NiO_3_ and a Mars-van-Krevelen type oxygen vacancy dominated one for La_0.9_Sr_0.1_Ni_0.5_Fe_0.5_O_3_. A similar switch in reaction mechanism has also been suspected for Mn-substituted La–Ni perovskites and we assume that a similar reaction mechanism also prevails at high Ti doping levels.^28^ All experiments yielded H_2_/CO ratios of 1 ± 0.05, verifying predominant DRM activity (Figure S6). Table S2, which compares the activity of different Ni perovskite samples, as well as Ni on Al_2_O_3_, reveals that LaNi_0.25_Ti_0.75_O_3_ after hydrogen reduction matches or even outperforms most comparable materials in terms of activity under comparable experimental conditions.

**Figure 9:**
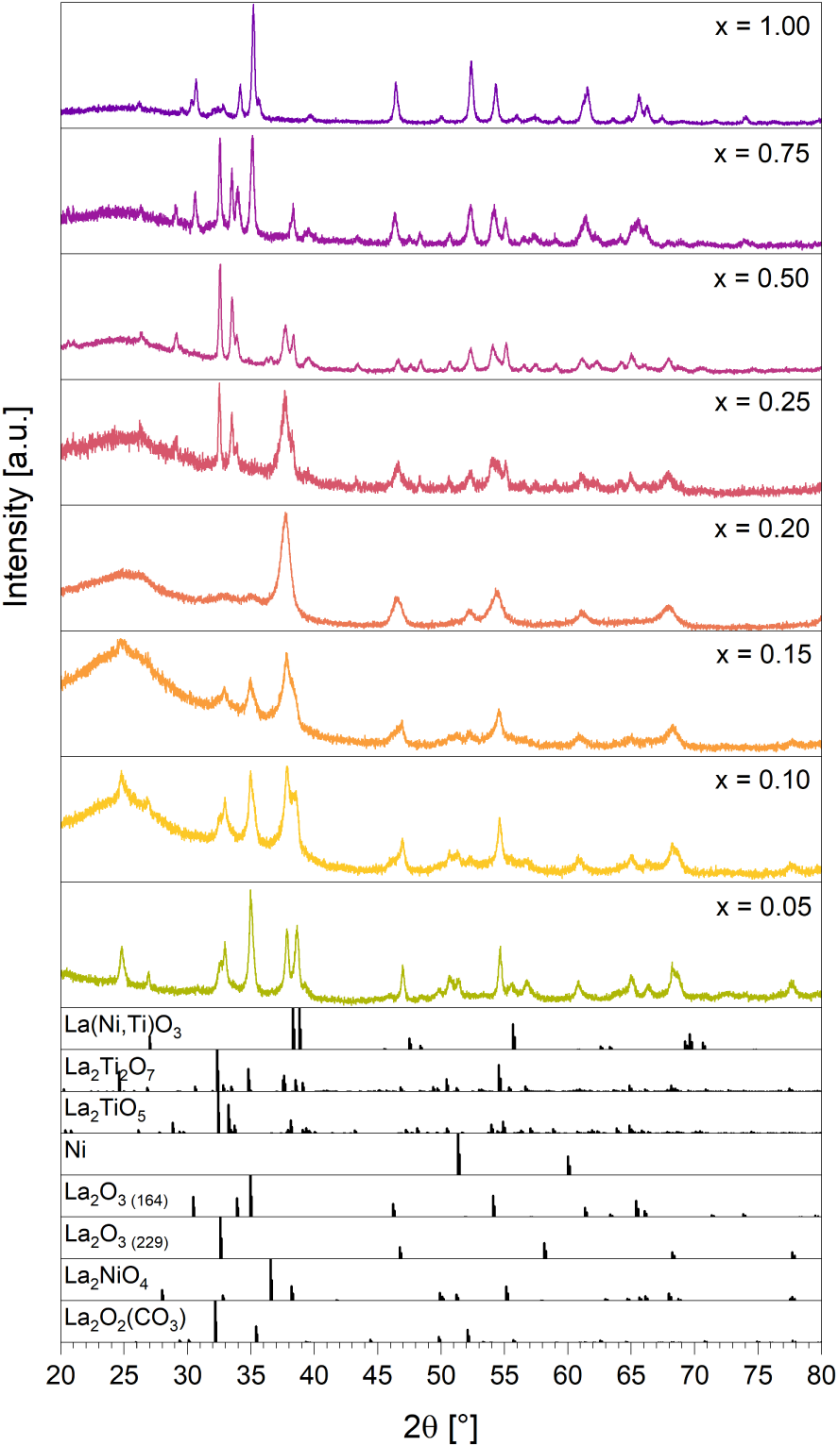
PXRD diffractograms of the LaNi_*x*_Ti_1–*x*_O_3_ samples after treatment in H_2_ followed by DRM. For the compositions of each sample see Table 7. References: La­(Ni,Ti)­O_3_ modified after^25^ and,^27^ La_2_Ti_2_O_7_,^26^ La_2_TiO_5_,^29^ Ni,^30^ La_2_O_3 (164)_,^31^ La_2_O_3 (229)_,^33^ La_2_NiO_4_,^44^ La_2_O_2_(CO_3_).^45^

To investigate the long-term DRM performance of the catalyst with the nominally lowest Ni content, LaNi_0.25_Ti_0.75_O_3_ was tested for 100 h time-on-stream in a DRM experiment at 800 °C after an initial H_2_ treatment. Upon reaching its maximum conversion rates after heating with 5 °C min^–1^ to 800 °C, both CO_2_ and CH_4_ conversion remain stable throughout the duration of the experiment (Figure S7). Similarly, the H_2_/CO ratio also remains persistently close to 1. Overall, LaNi_0.25_Ti_0.75_O_3_ shows no signs of deactivation and behaves similarly to LaNi_0.5_Ti_0.5_O_3_.^15^

PXRD after H_2_ treatment and subsequent DRM shows no major differences in composition compared to ones taken after only the H_2_ treatment (Figure 9). As discussed above, for x_Ni_ = 1.00 and 0.75 La_2_O_2_CO_3_ is formed, otherwise the phase compositions are almost identical (Table 7). Ni crystallite sizes are still in the same range of 10 nm −60 nm as before DRM. As the reduction temperature was chosen at 1000 °C, we can exclude major Ni particle sintering even at the highest DRM temperatures (800 °C).

**Table 7:** Calculated Compositions, Phases and Crystallite Sizes of the LaNi_*x*_Ti_1–*x*_O_3_ Samples After H_2_ Treatment and DRM as Derived from Quantitative Rietveld Analysis^a^

LaNi_x_Ti_1‑x_O_3_ Sample	Phase	Weight Fraction [wt %]	CrySize [nm]
x_Ni_ = 1.00	La_2_O_3 (164)_	30	55
	Ni	60	50
	La_2_O_3 (229)_	8	15
	La_2_O_2_CO_3_	6	65
x_Ni_ = 0.75	La_2_TiO_5_	58	75
	Ni	29	35
	La_2_O_3 (164)_	13	50
	La_2_O_2_CO_3_	*	-
x_Ni_ = 0.50	La_2_TiO_5_	56	70
	LaTiO_3_	25	30
	Ni	15	35
	La_2_NiO_4_	4	35
x_Ni_ = 0.25	La_2_TiO_5_	33	50
	LaTiO_3_	57	15
	Ni	10	25
x_Ni_ = 0.20	La_2_Ti_2_O_7_	45	10
	LaTiO_3_	46	15
	Ni	9	10
x_Ni_ = 0.15	La_2_Ti_2_O_7_	76	10
	LaTiO_3_	24	15
	Ni	*	-
x_Ni_ = 0.10	La_2_Ti_2_O_7_	75	20
	LaTiO_3_	21	20
	Ni	4	20
x_Ni_ = 0.05	La_2_Ti_2_O_7_	88	35
	LaTiO_3_	10	30
	Ni	2	-

a Phases marked with * are detectable, but fall below the quantification limit. The accuracy of all weight fractions is estimated to be ± 1–3 wt.%.

### 3.5 Influence of Ti Doping on the Surface Electronic Properties

The surface chemical and electronic properties in the as-calcined samples were determined by X-ray photolelectron spectroscopy (Figure 10). A full list of binding energies of different components and fit parameters is found in the SI. The overlap of the La 3d/Ni 2p region renders the interpretation less straightforward, but a shift of the Ni 2p component for x_Ni_ = 1.00 to x_Ni_ = 0.25 is evident. The respective binding energies are 854.7 and 853.7 eV for x_Ni_ = 1.00 and x_Ni_ = 0.25, respectively, indicating a gradual shift from Ni^3+^ to Ni^2+^.^46–50^ In pure LaNiO_3_, all Ni is found in the oxidation state 3+ to ensure charge neutrality. The shape of the complex La 3d region is significantly altered by the presence of Ni at high Ni contents, but shifts to the shape of La_2_O_3_ for x_Ni_ = 0.25.^51^ If Ti is substituted for Ni, Ti is found exclusively in the oxidation state 4+ for x_Ni_ = 0.75 (Ti^4+^ 2p_3/2_ at 458.5 eV), but increasing Ti amounts lead to more Ti in the oxidation state 3+ (Ti^3+^ 2p_3/2_ at 457.5 eV for x_Ni_ = 0.25).^52–55^ This is conceivable, as only a small amount of Ti^4+^ is required to compensate for the small amounts of Ni^2+^. The remaining Ti^3+^ is then again necessary for overall charge compensation of the perovskite. The O 1s region is significantly more complex – even for LaNiO_3_, three components are necessary to fit the measured data, which are assigned to lattice oxygen (O_lattice_) at 529.5 eV, vacancy-related oxygen (O_v_) at 530.3 eV and surface oxygen (O_surf_) at 532.1 eV, respectively.^47–49,56^ Increasing the Ti substitution from x_Ni_ = 0.75 to x_Ni_ = 0.50 and 0.25 increasingly populates the vacancy-related oxygen and simultaneously depopulates the surface oxygen species. This is a clear indication for the suggested switch to an oxygen-vacancy dominated reaction pathway at high Ti doping levels, when no La_2_O_2_CO_3_ formation occurs, in agreement with related studies on Fe-^43^ and Mn-doped^28^ LaNiO_3_. In addition, a Ti-related oxygen component appears at ≈528.6 eV, which gets more pronounced as the Ti content increases. We relate this feature to a Ti–O species, but there is no obvious relation to either a Ti^4+^ or Ti^3+^ component.

**Figure 10:**
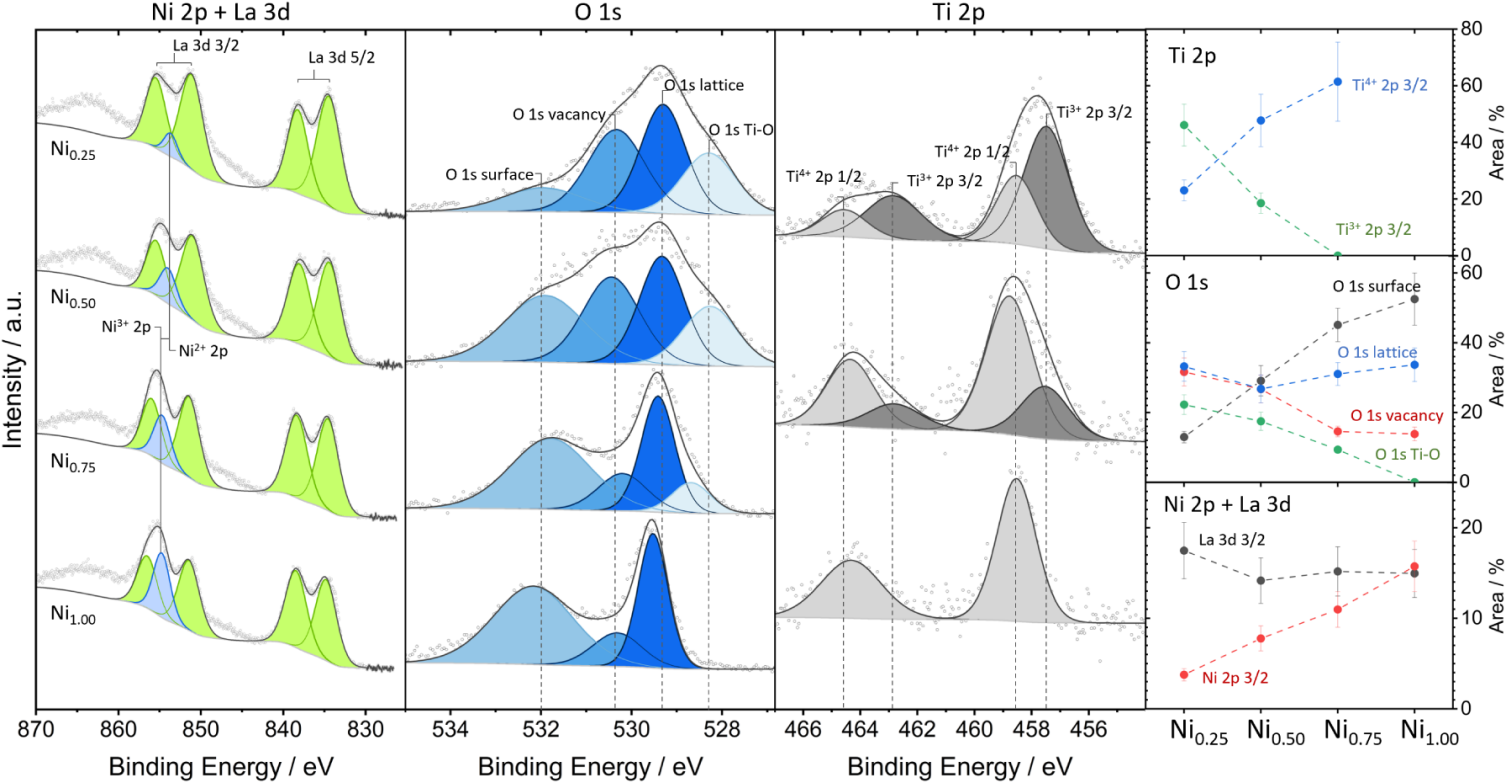
High-resolution X-ray photoelectron spectra of the La 3d/Ni 2p, O 1s and Ti 2p regions for selected LaNi_*x*_Ti_1–*x*_O_3_ samples with x_Ni_ = 1.00, 0.75, 0.50, and 0.25 in the as-calcined state. Spectra have been fitted by different components where applicable. The right Panel shows the semiquantitative variation of the areas of selected components as a function of Ti doping. Dashed lines between the composition points are shown as a guide-to-the-eye.

## 4 Conclusions

We have shown how the introduction of Ti into LaNiO_3_ influences the structure, reduction stability and catalytic properties in DRM operation. Ti doping of LaNiO_3_ significantly enhances the structural stability of LaNiO_3_ under reducing conditions. With increasing Ti doping levels, full or partial decomposition, leading to formation of La_2_O_3_, is only observed for LaNiO_3_ and x_Ni_ = 0.75. La_2_TiO_5_ formation is observed for x_Ni_ = 0.75, x_Ni_ = 0.50 and x_Ni_ = 0.25. For x_Ni_ < 0.25, formation of pyrochlore-type La_2_Ti_2_O_7_ occurs. Ni exsolution is not affected by Ti doping, as quantitative X-ray diffraction reveals that all nominally available Ni is exsolved during prereduction. In turn, Ti doping has a strong effect on the self-activation properties during DRM operation. Self-activation is strongly tied to reductive stability and thus, only possible for the lowest Ti doping levels of LaNiO_3_, x_Ni_ = 0.75 and 0.50. Below x_Ni_ = 0.50, the perovskite cannot be activated during DRM. Self-activation of LaNiO_3_ leads to full perovskite decomposition. For for x_Ni_ = 0.75, partial decomposition and associated formation of La_2_O_3_ is observed. For x_Ni_ = 0.50, the perovskite structure itself is maintained, although Ni exsolution occurs. As expected from the structural studies, the influence of Ti doping on the catalytic DRM properties, especially after hydrogen reduction to induce Ni exsolution, is considerable. The crucial Ti composition threshold is x_Ni_ = 0.20. At this composition, formation of La_2_Ti_2_O_7_ occurs, which has a detrimental effect on DRM properties. The more La_2_Ti_2_O_7_ is formed, the worse the catalytic DRM behavior. Mechanistic-wise, the formation or suppression of the La_2_O_2_CO_3_ phase is crucial at low Ti doping levels. With respect to self-activation, the DRM profiles for LaNiO_3_, and less pronounced for x_Ni_ = 0.75, exhibit characteristic drops in the CO_2_/CH_4_ conversion, which structurally match the redecomposition of intermediarily formed La_2_O_2_CO_3_. For x_Ni_ = 0.50 the same drop is not observed, as the entire activity is carried by exsolved Ni and the perovskite remains stable. The same drop is also observed for the same composition after hydrogen prereduction, in line with the observation of the presence of La_2_O_2_CO_3_ in spent catalyst PXRD patterns. Most importantly, our catalytic profiles, in conjunction with PXRD and XPS data, indicate that a change in the DRM pathway takes place between compositions of x_Ni_ = 0.75 and x_Ni_ = 0.50. While for the former, a literature-known oxycarbonate DRM mechanism still prevails,^2,3,6^ we suggest that for higher Ti doping levels, a second pathway via oxygen vacancies is followed, in line with O 1s XPS data and studies on similar Fe- and Mn-doped lanthanum–nickel based perovskites.^28,43^ At high Ni contents, La_2_O_2_CO_3_ prevails and no significant Ti influence is notable. Beneficial Ti influence is especially pronounced between x_Ni_ = 0.75 and _XNi_ = 0.25, where the Ti content is high enough to (partially) stabilize the reduced parent perovskite structure, but at the same time Ni exsolution is observed, leading to a Ni-perovskite interface potentially enabling a more vacancy-dominated pathway, as indicated in O 1s XP spectra. Our results also suggest, that with respect to LaNiO_3_, reducing the amount of Ni by successive Ti doping is possible down to x_Ni_ = 0.25 without significantly compromising the catalytic properties, making the LaNi_0.25_Ti_0.75_O_3_ perovskite the best catalyst in terms of balancing catalytic activity, long-term stability and economizing the Ni amount.

## Supplementary Material



## Data Availability

Data will be made available on a repository under doi: 10.48323/vtyq7-eap18.

## Notes

BET analysis; SEM/EDX analysis of the as-calcined states; Additional Rietveld refinements; SEM/EDX analysis of the hydrogen-reduced states; Temperature-programmed hydrogen reduction; H_2_/CO ratios and long-term experiment of LaNi_0.25_Ti_0.75_O; DRM properties of related Ni- and Co perovskite systems; Details of XPS analysis (PDF)
