# Supplementary material for: How Ti Doping Improves the Catalytic Methane Dry Reforming of Nanoporous Reduced LaNiO3 Perovskites
Source: ACS Appl Nano Mater. 2025 Nov 6;8(46):22339–51. doi: 10.1021/acsanm.5c04175 (PMC12645428; doi:10.1021/acsanm.5c04175)
Supplement: Supplementary file 1 [file an5c04175_si_001.pdf]

Supporting Information

How Ti Doping Improves the Catalytic Methane Dry Reforming of Nanoporous Reduced $LaNiO_3$ Perovskites

Thomas F. Winterstein,[1] Andreas Oss,[1] Christoph Malleier,[1] Asghar Mohammadi,[1] Bernhard Klötzer,[1] Stefan Stöber,[3] Volker Kahlenberg,[2] Simon Penner[1,*]

[1]Institute of Physical Chemistry, University of Innsbruck, Innrain 52c, A-6020 Innsbruck

[2]Institute of Mineralogy and Petrography, University of Innsbruck, Innrain 52d, A-6020 Innsbruck

[3]Institut für Geowissenschaften und Geographie, Martin-Luther Universität Halle, Von-Seckendorff – Platz 3, D-06120 Halle (Saale)

*Corresponding author: S. Penner, simon.penner@uibk.ac.at, +4351250758003

Keywords: single perovskite; Ti-doped $LaNiO_3$; in situ; $La_2Ti_2O_7$, thermogarvimetry, X-ray diffraction

Section A Details of materials synthesis

Table S1: Stoichiometric amounts of educts for the perovskite synthesis resulting in 3 g of sample each. Accuracy of weighed amounts: ± 0.0004 g.

x_{Ni}	$La(NO_3)_3$ □ 6 H_2O [g]	$Ni(NO_3)_2$ □ 6 H_2O [g]	$C_{12}H_{28}O_4Ti$ [g]	citric acid [g]	ethylene glycol [g]
1.00	5.2893	3.5521	0.0000	9.3873	6.0653
0.75	5.3482	2.6938	0.8776	9.4919	6.1329
0.50	5.4085	1.8161	1.7750	9.5988	6.2020
0.25	5.4701	0.9184	2.6928	9.7082	6.2727
0.20	5.4826	0.7364	2.8789	9.7304	6.2870
0.15	5.4952	0.5536	3.0658	9.7527	6.3014
0.10	5.5078	0.3699	3.2536	9.7751	6.3159
0.05	5.5205	0.1854	3.4423	9.7976	6.3304
0.00	5.5332	0.0000	3.6318	9.8201	6.3450

Section B BET analysis

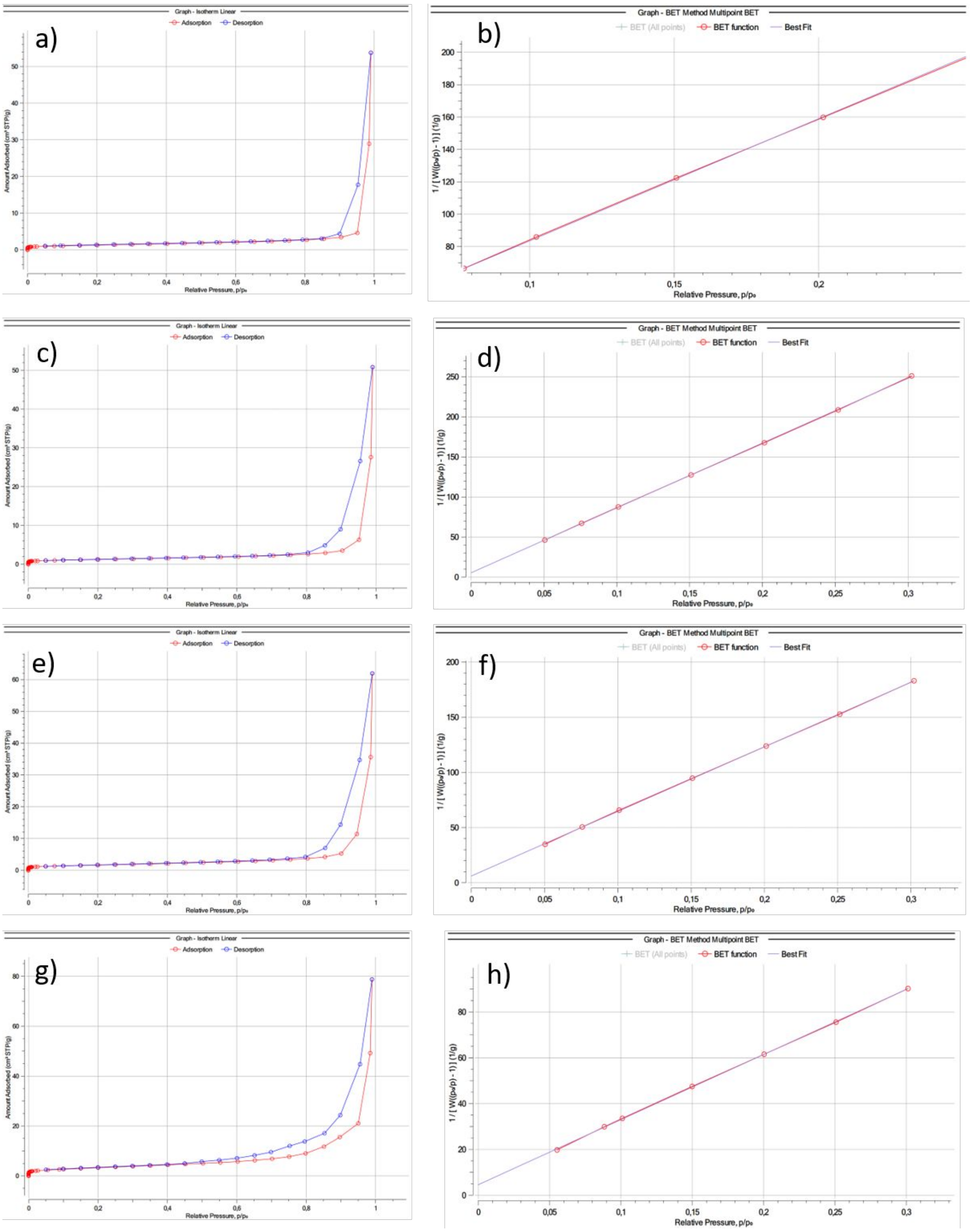

Figure S1: Adsorption-desorption isotherms and BET multi-point analysis for x_{Ni} = 1.00 (Panel a and b), x_{Ni} = 0.75 (Panel c and d), x_{Ni} = 0.50 (Panel e and f), x_{Ni} = 0.25 (Panel g and h)

Section C SEM/EDX analysis of the as-calcined states

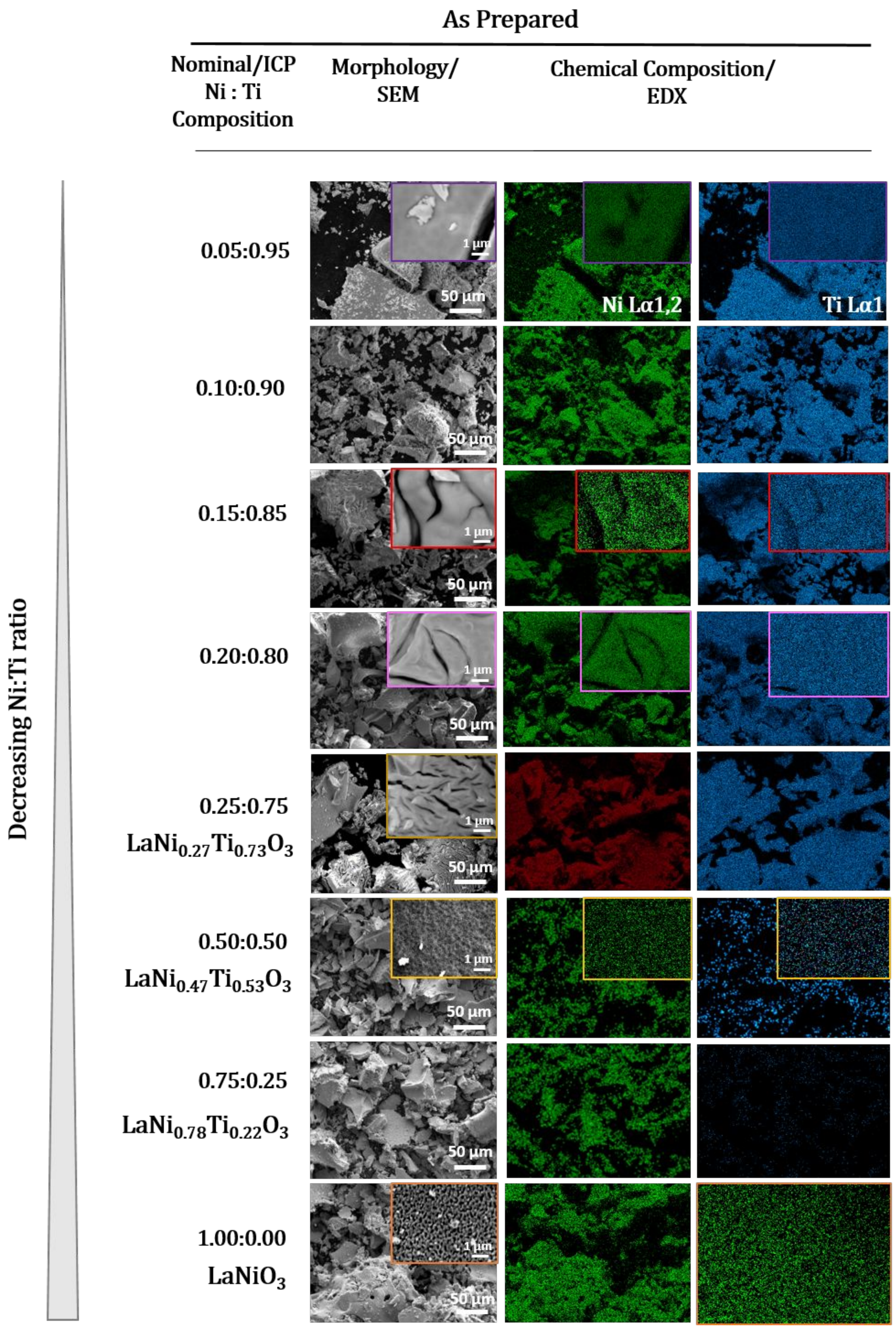

Figure S2: SEM/EDX characterization of the full $LaNi_xTi_{1-x}O_3$ compositional series and varying Ni/Ti ratio. The orange framed image in the right lower corner for $LaNiO_3$ is the Ni L map for the inset in the respective SEM image. For compositions, were a single phase has been determined, we are able to give a perovskite formula as derived from ICP analysis. For compositions $x_{Ni} \leq 0.20$, this is not possible due to the simultaneous presence of perovskite and pyrochlore phase.

Section D Additional Rietveld refinements

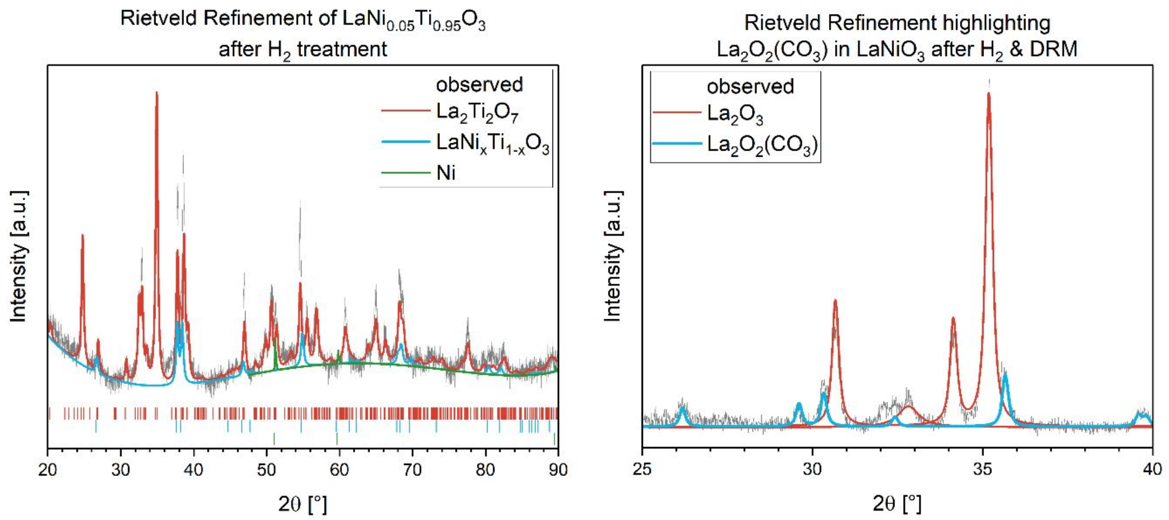

Figure S3: Rietveld refinement fits for $LaNi_{0.05}Ti_{0.95}O_3$ (left, after hydrogen treatment) and $La_2O_2CO_3$ in $LaNiO_3$ (right, after hydrogen treatment and DRM). Experimental conditions as in main manuscript.

Section E SEM/EDX analysis of the hydrogen-reduced states

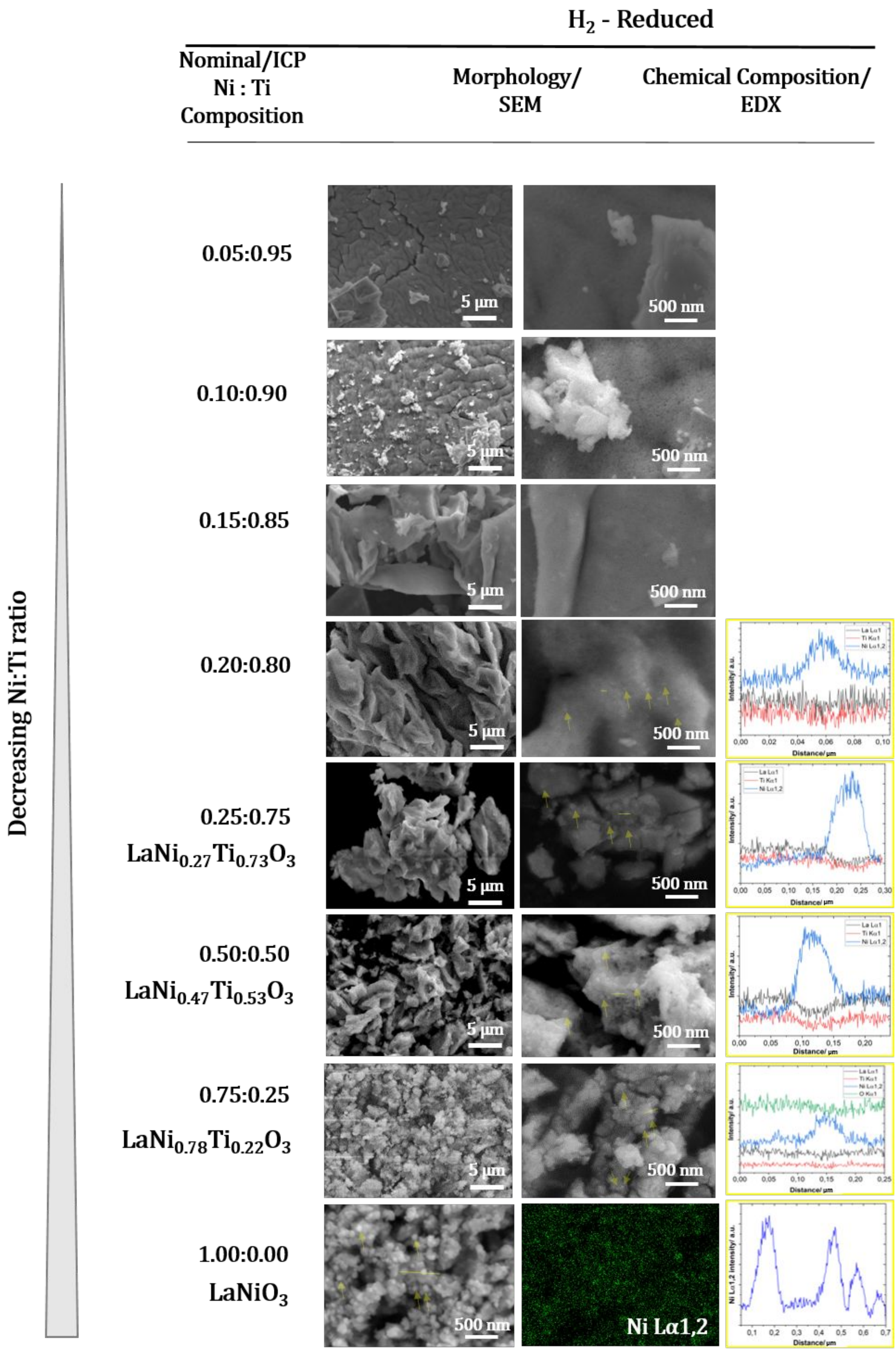

Figure S4: SEM/EDX characterization of the full $LaNi_xTi_{1-x}O_3$ compositional series and varying Ni/Ti ratio after hydrogen reduction at 1000 °C for 1 h. The yellow framed line profiles correspond to the indicated line progiles shown in the respective SEM images. Some exsolved Ni particles have been marked by yellow arrows. For compositions, were a single phase has been determined, we are able to give a perovskite formula as derived from ICP analysis. For compositions $x_{Ni} \leq 0.20$, this is not possible due to the simultaneous presence of perovskite and pyrochlore phase.

Section F Temperature-programmed hydrogen desorption

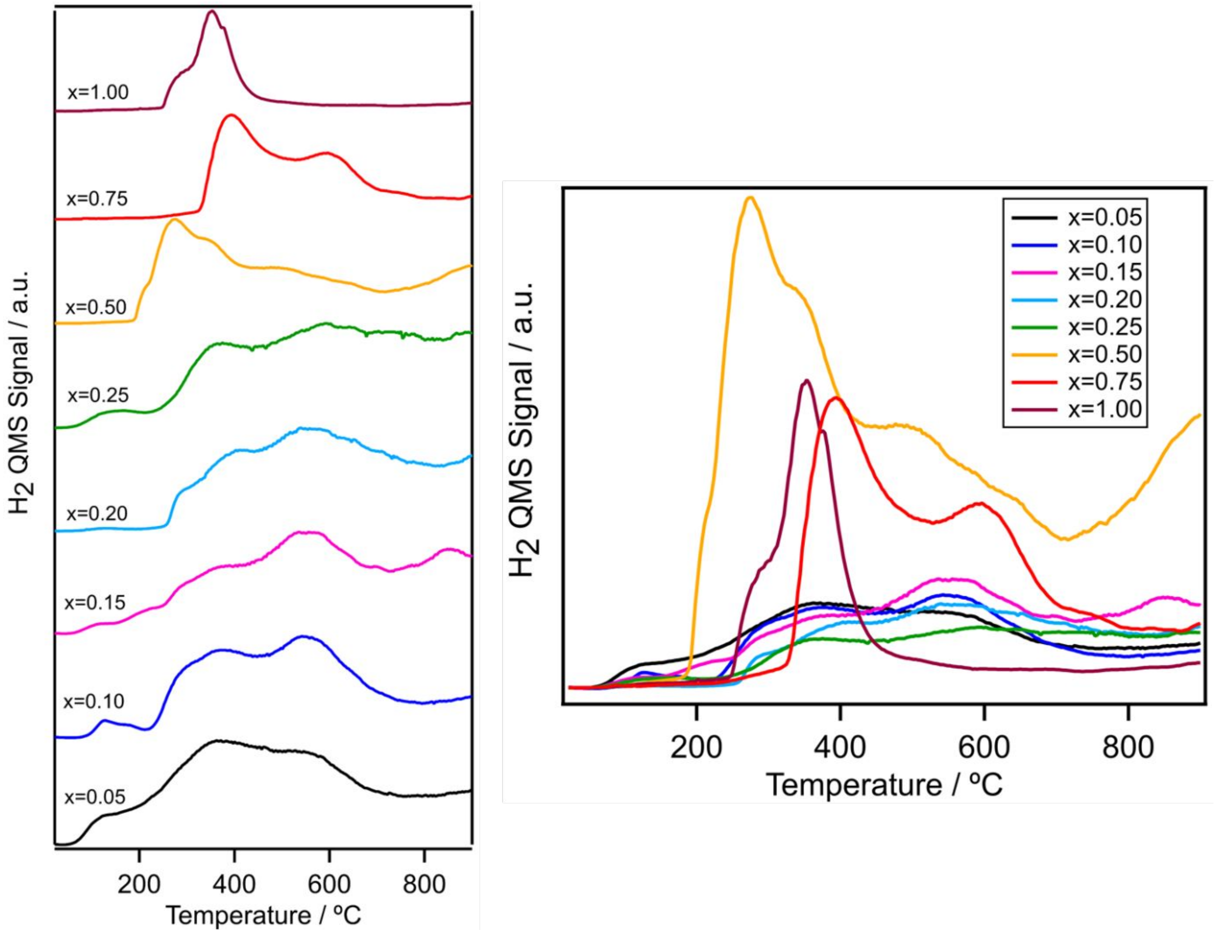

Figure S5: H_2 desorption profiles during temperature programmed desorption (TPD) tests carried out directly after H_2-TPR. The temperature program is identical to H_2-TPR. The base pressure of the system was 1□10^{-6} mbar. The right panel is plotted based on the same data as on the left panel for direct intensity comparison.

Section G H_2/CO ratios and long-term experiment of $LaNi_{0.25}Ti_{0.75}O_3$

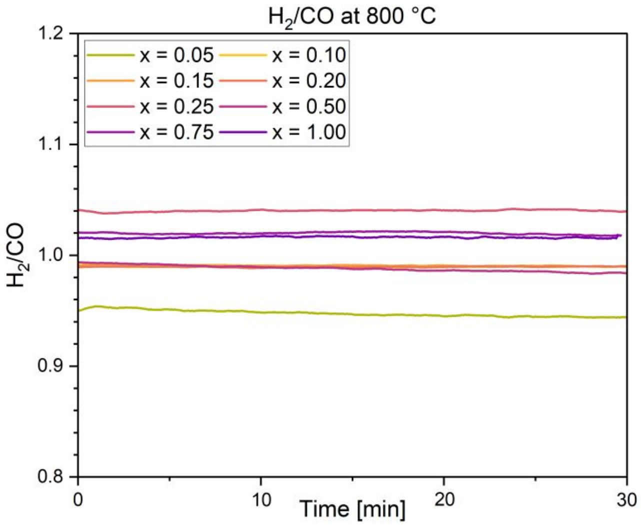

Figure S6: H_2/CO ratios in the isothermal section of the DRM experiments after hydrogen pre-reduction

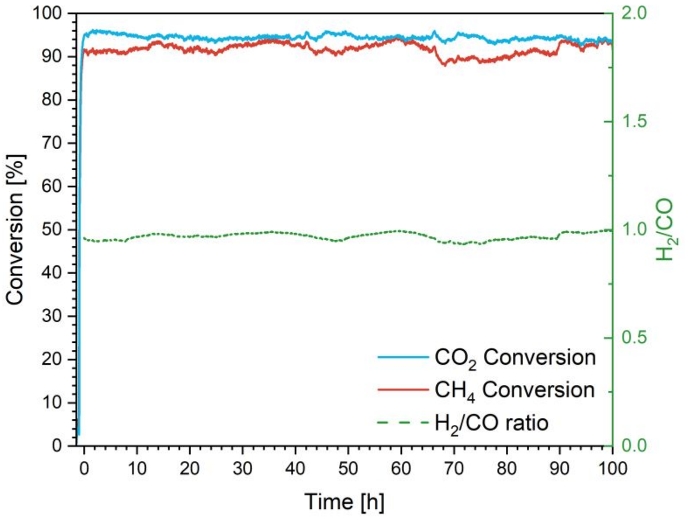

Figure S7: Conversion profiles and H_2/CO ratio of $LaNi_{0.25}Ti_{0.75}O_3$ after H_2 treatment and long-term DRM experiment at 800 °C for 100 h.

Table S2: DRM properties of related Ni- and Co perovskite systems

Catalyst Precursor	CO_2 Conversion [%]	CH_4 Conversion [%]	GHSV [$Lh^{-1}g_{cat}^{-1}$]	Rection Mixture	Temperature [°C]
Sr_2NiMoO_6 [15]	84.3	56.7	18	CH_4:CO_2:He 1:1:1	800
$Sr_2Ni_{0.5}Co_{0.5}MoO_6$ [15]	29.1	16.1	18	CH_4:CO_2:He 1:1:1	800
Ni on Al_2O_3 [15]	93.5	88.0	18	CH_4:CO_2:He 1:1:1	800
$LaNiO_3$ [15]	89.0	88.5	18	CH_4:CO_2:He 1:1:1	800
$LaNi_{0.5}Co_{0.5}O_3$ [1]	63	56	18	CH_4:CO_2 1:1	750
$LaNi_{0.5}Fe_{0.5}O_3$ [1]	85	85	12	CH_4:CO_2 1:1	750
$La_{1-x}Sr_xNiO_3$ [1]	71	85	-	CH_4:CO_2 1:1	700
$La_{1-x}Sr_xCoO_3$ [1]	56	63	24	CH_4:CO_2:Ar 1:1:8	800
$SrTi_{1-x}Ru_xO_3$ [1]	96	93	28.8	CH_4:CO_2:N_2 1:1:1	900
$SmCoO_3$ [1]	75	73	30	CH_4:CO_2 1:1	850
$NdCoO_3$ [1]	-	92.3	20	CH_4:CO_2 1:1	850

Section H Details of XPS analysis

Sample		Component	Pos. / eV BE	FWHM	Area / %
$LaNi_{0.25}Ti_{0.75}O_3$	Ni 2p + La 3d	La 3d 5/2 I	834.47	3.41	31.55
		La 3d 5/2 II	838.25	2.99	21.06
		La 3d 3/2 I	851.13	3.27	26.17
		La 3d 3/2 II	855.45	2.94	17.47
		Ni 2p 3/2	853.67	1.91	3.76
	O 1s	O 1s Ni-O	529.30	1.18	33.21
		O 1s La-O	530.33	1.48	31.62
		O 1s Ni-OH	531.96	2.09	12.94
		O 1s Ti-O	528.29	1.40	22.24
	Ti 2p	Ti^{3+} 2p 3/2	457.48	1.85	46.10
		Ti^{3+} 2p 1/2	462.84	2.30	20.56
		Ti^{4+} 2p 3/2	458.53	1.65	23.06
		Ti^{4+} 2p 1/2	464.56	1.85	10.28

Sample		Component	Pos. / eV BE	FWHM	Area / %
$LaNi_{0.50}Ti_{0.50}O_3$	Ni 2p + La 3d	La 3d 5/2 I	834.42	3.27	28.42
		La 3d 5/2 II	838.06	3.27	24.19
		La 3d 3/2 I	850.99	3.28	25.46
		La 3d 3/2 II	855.43	2.78	14.16
		Ni 2p 3/2	853.99	2.36	7.77
	O 1s	O 1s Ni-O	529.33	1.18	26.75
		O 1s La-O	530.44	1.48	26.70
		O 1s Ni-OH	531.91	2.09	29.08
		O 1s Ti-O	528.26	1.40	17.46
	Ti 2p	Ti^{3+} 2p 3/2	457.53	1.98	18.54
		Ti^{3+} 2p 1/2	462.81	2.20	9.89
		Ti^{4+} 2p 3/2	458.77	1.93	47.74
		Ti^{4+} 2p 1/2	464.35	1.98	23.83

Sample		Component	Pos. / eV BE	FWHM	Area / %
$LaNi_{0.75}Ti_{0.25}O_3$	Ni 2p + La 3d	La 3d 5/2 I	834.58	3.07	26.80
		La 3d 5/2 II	838.38	3.07	24.15
		La 3d 3/2 I	851.43	2.96	22.92
		La 3d 3/2 II	855.96	2.76	15.17
		Ni 2p 3/2	854.69	2.39	10.97
	O 1s	O 1s Ni-O	529.41	0.88	31.02
		O 1s La-O	530.20	1.28	14.54
		O 1s Ni-OH	531.79	2.09	45.10
		O 1s Ti-O	528.68	1.00	9.33
	T	Ti^{4+} 2p 3/2	458.53	1.62	61.42

		Component	Pos. / eV BE	FWHM	Area / %
		Ti^{4+} 2p 1/2	464.31	2.49	38.58
$LaNiO^3$	Ni 2p + La 3d	**Component**	**Pos. / eV BE**	**FWHM**	**Area / %**
		La 3d 5/2 I	834.81	3.04	24.75
		La 3d 5/2 II	838.46	3.04	23.54
		La 3d 3/2 I	851.44	2.99	21.04
		La 3d 3/2 II	856.50	2.84	14.95
		Ni 2p 3/2	854.74	2.42	15.73
	O 1s	O 1s Ni-O	529.53	0.78	33.67
		O 1s La-O	530.32	1.28	13.81
		O 1s Ni-OH	532.17	2.09	52.52
